# Central Role for MCP-1/CCL2 in Injury-Induced Inflammation Revealed by *In Vitro*, *In Silico*, and Clinical Studies

**DOI:** 10.1371/journal.pone.0079804

**Published:** 2013-12-03

**Authors:** Cordelia Ziraldo, Yoram Vodovotz, Rami A. Namas, Khalid Almahmoud, Victor Tapias, Qi Mi, Derek Barclay, Bahiyyah S. Jefferson, Guoqiang Chen, Timothy R. Billiar, Ruben Zamora

**Affiliations:** 1 Department of Surgery, University of Pittsburgh School of Medicine, Pittsburgh, Pennsylvania, United States of America; 2 Department of Computational and Systems Biology, University of Pittsburgh School of Medicine, Pittsburgh, Pennsylvania, United States of America; 3 Center for Inflammation and Regenerative Modeling, McGowan Institute for Regenerative Medicine, Pittsburgh, Pennsylvania, United States of America; 4 Joint Carnegie Mellon University – University of Pittsburgh Ph.D. Program in Computational Biology, Pittsburgh, Pennsylvania, United States of America; 5 Department of Neurology and Pittsburgh Institute for Neurodegenerative Diseases, University of Pittsburgh, Pittsburgh, Pennsylvania, United States of America; 6 Sports Medicine and Nutrition, University of Pittsburgh School of Medicine, Pittsburgh, Pennsylvania, United States of America; 7 Department of Anesthesiology, Shanghai Tenth People's Hospital, Tongji University School of Medicine, Shanghai, China; Rutgers University, United States of America

## Abstract

The translation of *in vitro* findings to clinical outcomes is often elusive. Trauma/hemorrhagic shock (T/HS) results in hepatic hypoxia that drives inflammation. We hypothesize that *in silico* methods would help bridge *in vitro* hepatocyte data and clinical T/HS, in which the liver is a primary site of inflammation. Primary mouse hepatocytes were cultured under hypoxia (1% O_2_) or normoxia (21% O_2_) for 1–72 h, and both the cell supernatants and protein lysates were assayed for 18 inflammatory mediators by Luminex™ technology. Statistical analysis and data-driven modeling were employed to characterize the main components of the cellular response. Statistical analyses, hierarchical and k-means clustering, Principal Component Analysis, and Dynamic Network Analysis suggested MCP-1/CCL2 and IL-1α as central coordinators of hepatocyte-mediated inflammation in C57BL/6 mouse hepatocytes. Hepatocytes from MCP-1-null mice had altered dynamic inflammatory networks. Circulating MCP-1 levels segregated human T/HS survivors from non-survivors. Furthermore, T/HS survivors with elevated early levels of plasma MCP-1 post-injury had longer total lengths of stay, longer intensive care unit lengths of stay, and prolonged requirement for mechanical ventilation vs. those with low plasma MCP-1. This study identifies MCP-1 as a main driver of the response of hepatocytes *in vitro* and as a biomarker for clinical outcomes in T/HS, and suggests an experimental and computational framework for discovery of novel clinical biomarkers in inflammatory diseases.

## Introduction

Among many other functions, the liver plays a critical role in inflammation and innate immunity, processes that are controlled by multiple cell types including hepatocytes, Kupffer cells, and other non-parenchymal cells. Although at least 15 different cell types can be found in normal liver [1], hepatocytes constitute the largest pool of parenchymal cells, comprising approximately 60–80% of the total liver cells [1], [2]. Inflammatory conditions such as ischemia/reperfusion (I/R) and post-trauma hemorrhagic shock (T/HS) are associated with liver hypoxia [3], [4]. It is now accepted that hypoxia is not merely an outcome of the inflammatory response, but rather is a key driver of the development of inflammation through the regulation of O_2_-dependent signal transduction and gene expression [5], [6].

Mathematical and computational (*in silico*) methods have emerged as adjuncts to *in vitro* and *in vivo* studies of acute inflammation [7]. For example, we have recently applied both mechanistic and data-driven computational modeling to help define the dynamic, multi-dimensional inflammatory response to T/HS *in vivo* [8]–[12]. The goal of the present study was to determine if combined *in vitro*/*in silico* studies could help elucidate key hepatic inflammatory mediators relevant to human T/HS. This study identifies the chemokine Monocyte Chemoattractant Protein-1 (MCP-1/CCL2) as a main driver of the response of hepatocytes *in vitro* and as a biomarker for organ damage in clinical settings of T/HS, and, more generally, suggests a pathway for combined experimental and computational studies to facilitate the discovery of novel clinical biomarkers of inflammation.

## Results

### MCP-1 is a central component of the dynamic, multi-dimensional response of hepatocytes to cell stress

**Figure 1:**
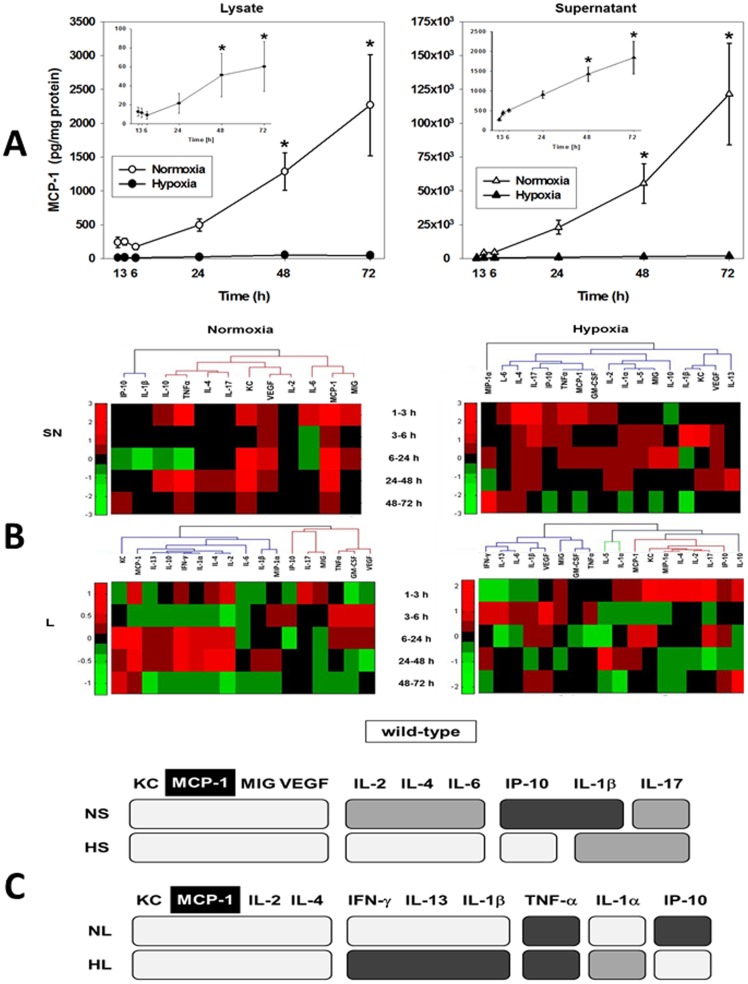
Inflammatory mediator production by primary mouse hepatocytes and meta-clustering analysis. Freshly isolated hepatocytes from C57BL/6 (wild-type) mice were cultured under normoxic (control, 21% O_2_, open symbols) or hypoxic (1% O_2_, closed symbols) conditions for 1–72 h as described in the *Materials and Methods*. Both lysates and supernatants were assayed for 18 mouse inflammatory mediators using the Luminex xMAP technology and the measurements were normalized for protein content as indicated. (**A**) MCP-1 expression and release in primary mouse hepatocytes (mean ± SEM, n = 4–8 independent experiments, analyzed by Two-Way ANOVA followed by the Holm-Sidak *post-hoc* test, *P<0.001, normoxia vs. hypoxia within a specific time point). The insets show the levels of MCP-1 in hypoxia samples. (**B**) Hierarchical clustering over fold changes in wild-type hepatocytes (normoxia vs. hypoxia): Fold change values for each inflammatory mediator are represented in heat maps, ranging from large negative (green) to large positive values (red). No changes (zero values) changes are represented in black (see *Materials and Methods*). (**C**) Comparison between meta-clustering analysis outcomes in normoxia and hypoxia (see *Materials and Methods*). The shading of the boxes indicates the grouping of mediators that exhibited the same segregation pattern across all methods. For each experimental condition (NL, HL, NS and HS), only the mediators appearing in each consensus are shown. For comparison between experimental conditions only mediators common to both consensuses are shown. The consensus clusters characterize the cellular response and were derived from hierarchical clustering (mediators with similar dynamic trajectories) and from PCA (mediators with the strongest covariance with other mediators).

To assess the response of hepatocytes to hypoxia, primary wild-type mouse hepatocytes were subjected to 1% O_2_ for 1–72 h, and 18 mouse cytokines were measured in both the supernatant and whole-cell lysate. Hepatocytes cultured under normoxic (21% O_2_) conditions served as controls. One-way ANOVA showed that, in normoxic hepatocytes, MCP-1, KC, and IP-10 (in lysates) and MCP-1, KC, and MIG (in supernatants) were altered significantly (**Table 1**). In hypoxic hepatocytes, the significantly altered mediators were MCP-1, MIG, IL-1α, IL-1β, IL-10, and IL-13 (in lysates) and MCP-1, IP-10, IL-1α, and VEGF (in supernatants). Thus, **MCP-1** was the only mediator that exhibited significant changes in all four conditions examined, as shown in **Fig. 1A**.

**Table 1:** Significance levels (*P*<0.05) for production and release of inflammatory mediators from wild-type mouse hepatocytes cultured under normoxic or hypoxic conditions (1–72 h) as determined by one-way ANOVA.

	Normoxia		Hypoxia^a^	
	Lysate	Supernatant	Lysate	Supernatant
GM-CSF	0.721	1.0	0.753	0.086
IFN-γ	0.105	1.0	0.452	1.0
**IL-1α**	0.591	1.0	**0.023**	**0.007**
**IL-1β**	0.559	0.664	**0.026**	0.886
IL-2	0.126	0.929	0.240	0.919
IL-4	0.901	0.942	0.935	0.656
IL-5	1.0	1.0	0.123	0.101
IL-6	0.091	0.914	0.223	0.059
**IL-10**	0.346	0.947	**0.035**	0.832
**IL-13**	0.601	1.0	**0.002**	0.650
IL-17	0.980	0.921	0.958	0.999
**IP-10**	**0.045**	0.513	0.135	**0.046**
**KC**	**0.002**	**<0.001**	0.374	0.283
**MCP-1**	**0.001**	**<0.001**	**0.003**	**<0.001**
**MIG**	0.150	**0.002**	**0.016**	0.177
MIP-1α	0.636	0.416	0.081	0.543
TNF-α	0.987	0.685	0.056	0.999
**VEGF**	0.271	**<0.001**	0.073	**<0.001**

a For hypoxia time courses, the analysis was performed using as baseline the respective concentration values for normoxia at 1 h.

### Hierarchical Clustering and Principal Component Analysis reveal key differences in inflammatory mediator production/release in the hepatocyte response to cell stress

In order to address the question of which groups of mediators exhibit similar dynamics in normoxic and hypoxic hepatocytes, we performed hierarchical clustering on fold changes for each mediator at each pair of consecutive time points. We examined both raw values and fold change values for all mediators over each time interval. Each inflammatory mediator's values over time made up its dynamic pattern of expression and release. To identify those inflammatory mediators that showed similar production or secretion behavior, these patterns were then compared and grouped using hierarchical clustering, with inflammatory mediators in the resulting dendrogram ordered according to their membership in the clusters that emerged (**Fig. 1B**).

We also sought to determine the subset of mediators that evolve dynamically in a manner that is most characteristic of the response to normoxia or hypoxia, respectively. Inflammatory mediators that have similar patterns of covariance have similar loadings onto principal components, and so would be expected to project in a similar direction in principal component space. We therefore used the angle of an inflammatory mediator's projection into principal component space as the basis of a distance metric in k-means clustering (**Figures S1, S2, S3, S4)**. This analysis suggested that, despite an overlap in common mediators, k-means clustering could differentiate normoxia from hypoxia and lysates from supernatants. Furthermore, MCP-1 and KC were generally grouped together and were the predominant mediators along the first principal component.

### A predominant role for MCP-1, KC, and IL-1α in the hepatocyte response to stress inferred from Dynamic Network Analysis

We sought to determine the consensus of the three aforementioned, independent clustering experiments. If the cluster memberships were similar, then those mediators that did not segregate consistently across different clusterings could be treated as noise. In contrast, those inflammatory mediators that consistently segregated together were considered as mediators exhibiting similar or correlated dynamic trajectories. We first calculated an Adjusted Rand Index (ARI) [13]–[16] to quantify the degree to which any two clustering results were in agreement. This comparison (see **Figures S9, S10, S11, S12**) resulted in a “consensus clustering” showing the inflammatory mediators that exhibited the same segregation pattern across all analysis methods, suggesting that MCP-1 and KC segregate together across all experimental conditions (**Fig. 1C**).

**Figure 2:**
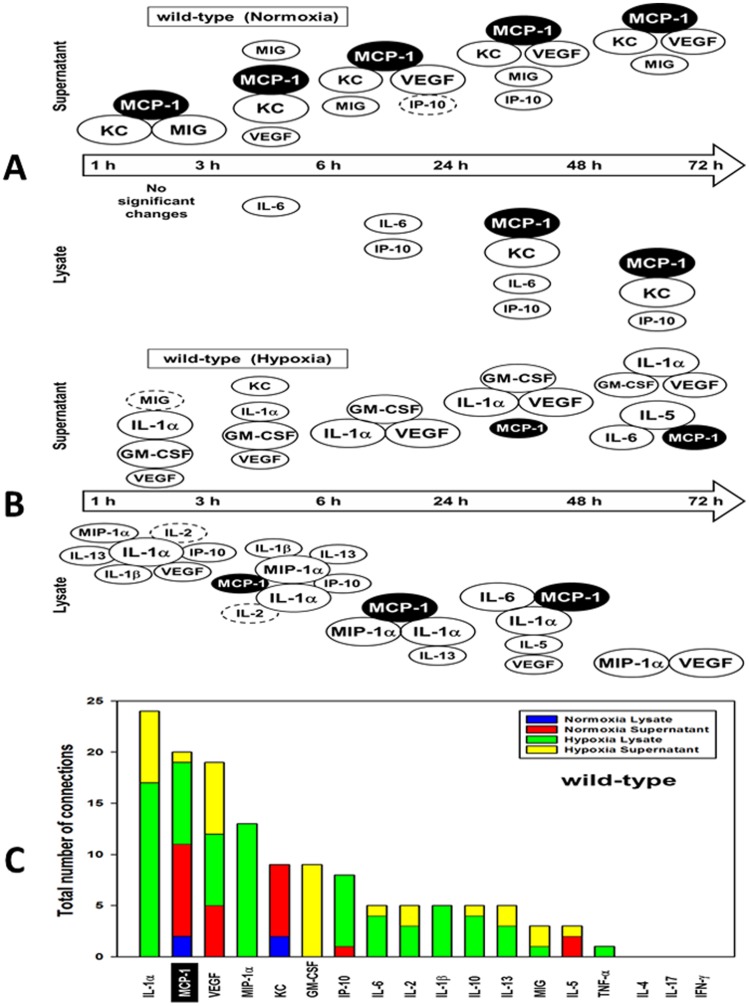
Dynamic Network Analysis (DyNA) of inflammatory mediators produced by normoxic and hypoxic mouse hepatocytes. Primary hepatocytes from wild-type mice were cultured under normoxic or hypoxic conditions (1–72 h) followed by measurement of inflammatory mediators in both lysates and supernatants and lysates as described in the *Materials and Methods*. After data normalization, DyNA during each of the following five time frames: 1–3 h, 3–6 h, 6–24 h, 24–48 h, and 48–72 h was performed for both lysates and supernatants as indicated. Panels show a summary of the DyNAs representing the most connected inflammatory mediator “nodes” for both normoxia (**A**) and hypoxia (**B**) lysates and supernatants. Each mediator's node size is proportional to the number of connections it has in a given time interval. (**C**) Stacked bars representing the total number of connections for each inflammatory mediator over all time intervals.

This meta-clustering analysis suggested groups of inflammatory mediators with similar dynamic patterns. We next performed Dynamic Network Analysis (DyNA) [12] to suggest mediators that appear to be “hubs” (or most connected to other mediators). (**Figs. 2A–B** **; Figure S17**). The total number of dynamic network connections is shown in **Fig. 2C**. Based on DyNA, MCP-1 and KC were the most connected mediators across all time points in normoxia supernatants, and at 24–72 h in normoxia lysates (**Fig. 2A**). In hypoxia, the most connected inflammatory mediators were IL-1α and VEGF in supernatants and IL-1α and MIP-1α in lysates (**Fig. 2B**). Overall, the most connected inflammatory mediators were MCP-1 and IL-1α, with MCP-1 being the only highly-connected node present in all experimental conditions examined (**Fig. 2C**).

### Inflammatory dynamics and networks are disrupted in MCP-1^−/−^ hepatocytes

**Figure 3:**
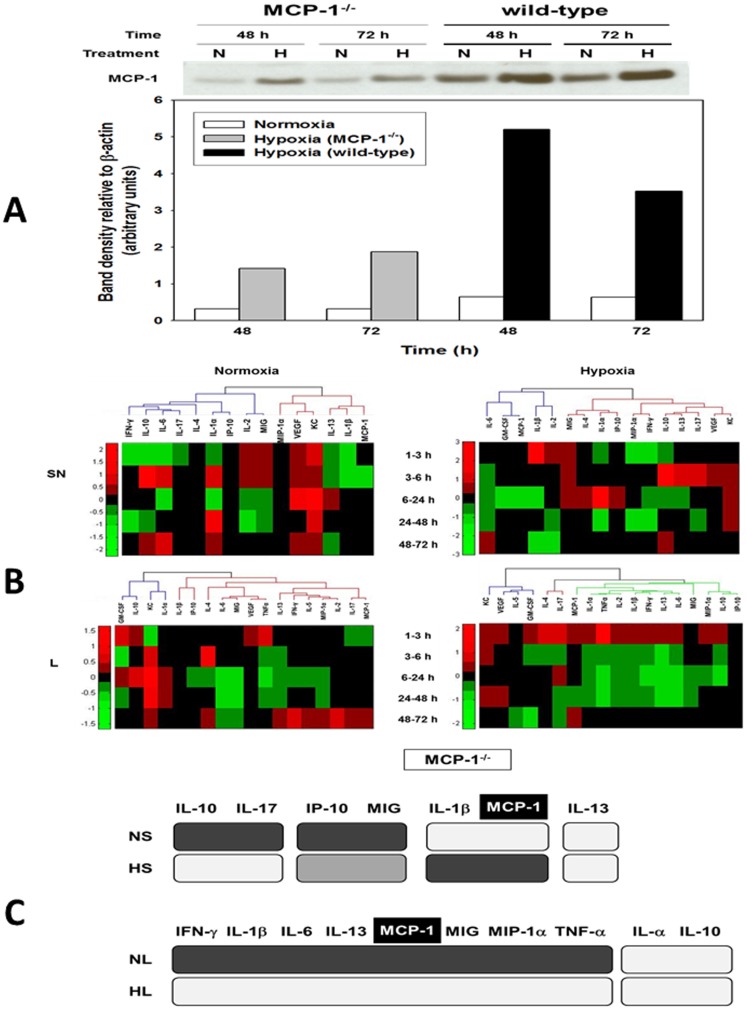
MCP-1 is a central component of the dynamic, multi-dimensional response of hepatocytes to cell stress. Primary hepatocytes from wild-type and MCP-1^−/−^ mice were cultured under normoxic (N) or hypoxic (H) conditions for 1–72 h, followed by Luminex™ analysis of 18 inflammatory mediators in both the supernatant (SN) and whole-cell lysate (L). The measurements were normalized and hierarchical and k-means clustering was performed as described in the *Materials and Methods*. (**A**) A representative Western blot showing MCP-1 protein expression in cell lysates from normoxic (N) and hypoxic (H) MCP-1^−/−^ and wild-type mouse hepatocytes (48 and 72 h) and densitometric analysis as described in the *Materials and Methods*. (**B**) Hierarchical clustering over fold changes in MCP-1^−/−^ hepatocytes (normoxia vs. hypoxia): Fold change values for each inflammatory mediator ranging from large negative (green) to large positive values (red) are shown. No fold changes (zero values) are represented in black. (**C**) Comparison between meta-clustering analysis outcomes in normoxia and hypoxia (see *Materials and Methods*). The shading of the boxes indicates the grouping of mediators that exhibited the same segregation pattern across all methods. For each experimental condition (NL, HL, NS and HS), only the mediators appearing in each consensus are shown. For comparison between experimental conditions only mediators common to both consensuses are shown.

Hypothesizing a central role for MCP-1 in the response of mouse hepatocytes to cell stress based on the above analyses, we repeated these experiments using hepatocytes from MCP-1^−/−^ mice. We first sought to verify that MCP-1 was absent in MCP-1^−/−^ mice. As shown in **Fig. 3A**, normoxic hepatocytes from MCP-1^−/−^ mice did not express MCP-1 by Western blot, while a weaker band of approx. 30 kDa was detected in the lysates from hypoxic cells. In contrast, MCP-1 protein expression was significantly higher in cell lysates of both normoxic and hypoxic hepatocytes from wild-type mice at 48 and 72 h (**Fig. 3A**). This analysis suggested the low, but detectable, presence of a protein with MCP-1-like characteristics in the nominally MCP-1^−/−^ mice.

One-way ANOVA (**Table 2**) suggested that, in normoxic MCP-1^−/−^ hepatocytes, only MIG, KC, and IL-6 (in lysates) and KC, IL-1β, and VEGF (in supernatants) were altered significantly. In hypoxic MCP-1^−/−^ hepatocytes, MIP-1α, IL-1α, IL-1β, IL-2, IL-6, IL-10, IL-13, IFN-γ, and TNF-α (in lysates) and KC, IL-1α, and VEGF (in supernatants) were altered significantly. Hierarchical clustering defined different networks and modules of mediators in MCP-1^−/−^ hepatocytes (**Fig. 3B**) as compared to wild type cells (**Fig. 1B**). Meta-clustering (**Figures S5, S6, S7, S8**) suggested that IL-1β and MCP-1 clustered together across all 4 experimental conditions in MCP-1^−/−^ hepatocytes (**Fig. 3C**).

**Table 2:** Significance levels (*P*<0.05) for production and release of inflammatory mediators from MCP-1^−/−^ mouse hepatocytes cultured under normoxic or hypoxic conditions (1–72 h) as determined by one-way ANOVA.

	Normoxia		Hypoxia^a^	
	Lysate	Supernatant	Lysate	Supernatant
GM-CSF	0.918	0.535	0.239	0.423
**IFN-γ**	0.897	0.371	**<0.001**	0.530
**IL-1α**	0.869	0.249	**0.002**	**0.034**
**IL-1β**	0.748	**0.031**	**0.008**	0.369
**IL-2**	0.978	0.332	**0.006**	0.282
IL-4	0.982	0.669	0.978	0.054
IL-5	0.757	1.0	0.379	1.0
**IL-6**	**<0.001**	0.475	**<0.001**	0.403
**IL-10**	0.439	0.696	**<0.001**	0.570
**IL-13**	0.191	0.888	**<0.001**	0.978
IL-17	0.992	0.995	0.960	0.945
IP-10	0.783	0.572	0.925	0.518
**KC**	**<0.001**	**<0.001**	0.234	**0.009**
MCP-1	0.823	0.535	0.573	0.782
**MIG**	**0.003**	0.761	0.609	0.068
**MIP-1α**	0.363	0.078	**0.009**	0.653
**TNF-α**	0.833	1.0	**0.002**	1.0
**VEGF**	0.599	**<0.001**	0.242	**0.019**

a For hypoxia time courses, the analysis was performed using as baseline the respective concentration values for normoxia at 1 h.

### Dynamic Network Analysis suggests altered networks of inflammatory mediators in MCP-1^−/−^ hepatocytes

**Figure 4:**
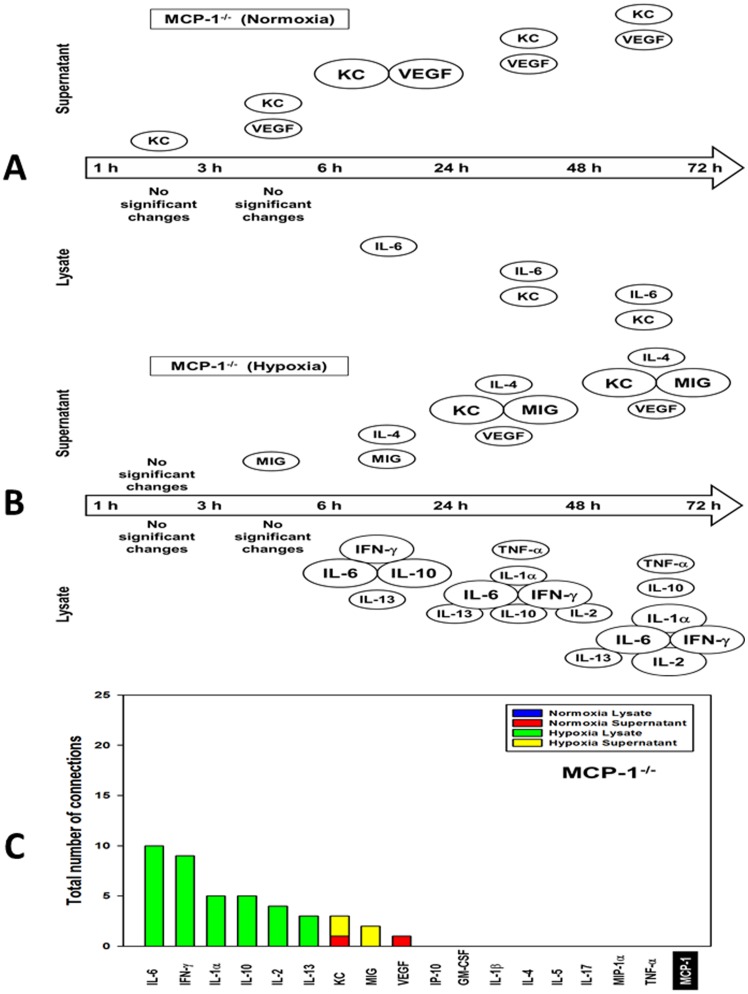
Dynamic Network Analysis (DyNA) of inflammatory mediators produced by normoxic and hypoxic mouse hepatocytes isolated from MCP-1^−/−^ mice. Primary hepatocytes isolated from MCP-1^−/−^ mice were cultured under normoxic or hypoxic conditions (1–72 h) followed by measurement of inflammatory mediators in both lysates and supernatants as described in the *Materials and Methods*. After data normalization, DyNA was performed for both lysates and supernatants during each of the following five time frames: 1–3 h, 3–6 h, 6–24 h, 24–48 h, and 48–72 h as indicated. Panels show a summary of the DyNAs representing the principal inflammatory mediator “nodes” for both normoxia (**A**) and hypoxia (**B**) lysates and supernatants. (**C**) Stacked bars representing the total number of connections for each inflammatory mediator over all time intervals.

We next carried out DyNA of lysates and supernatants from normoxic and hypoxic MCP-1^−/−^ hepatocytes (**Figs. 4A–C** **; Figure S18**). This analysis suggested a significant decrease in the number of connections (**Figs. 4A–B**) in both lysates and supernatants of normoxic cells, and in hypoxia supernatants, as compared to wild-type hepatocytes (see **Figs. 2A–B**). In supernatants of normoxic MCP-1^−/−^ hepatocytes, KC was the most significantly changed mediator (though with nearly zero connections across all time points; **Fig. 4A**), whereas in supernatants of hypoxic MCP-1^−/−^ hepatocytes this chemokine was a central node with one connection only at 24–72 h (**Fig. 4B**). In lysates from normoxic MCP-1^−/−^ hepatocytes, IL-6 was the most significantly changed mediator, but with zero connections (**Fig. 4A**). However, this cytokine was the most connected mediator (along with IFN-γ) in hypoxia lysates at all-time intervals after 6 h (**Fig. 4B**). DyNA suggested a clear reduction in the total number of dynamic connections among inflammatory mediators in MCP-1^−/−^ cells, with IL-6 being the most connected mediator (but only in hypoxia lysates; **Fig. 4C**).

### IL-6 expression and release is reduced in MCP-1^−/−^ hepatocytes

**Figure 5:**
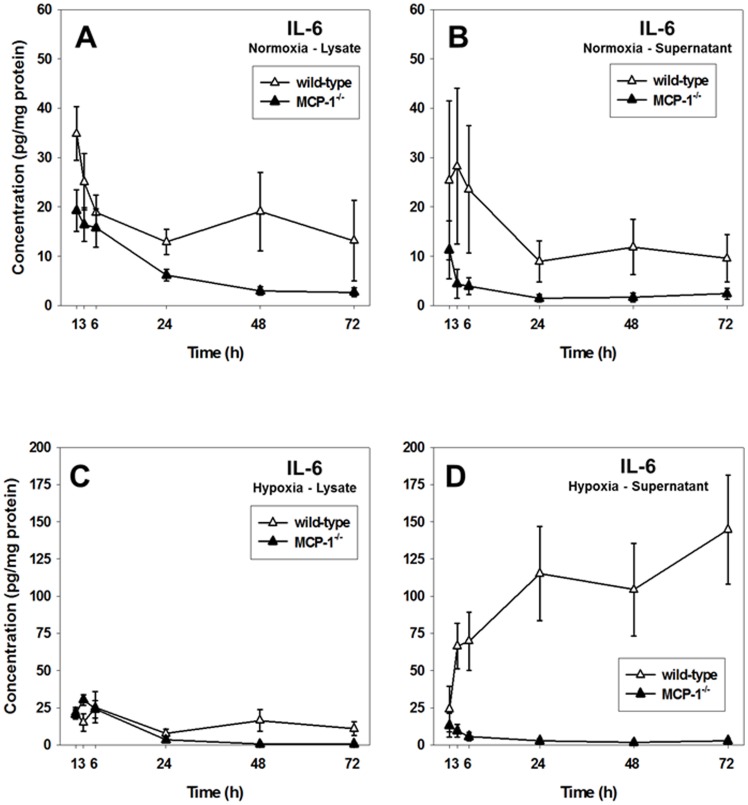
Time-dependent expression and release of IL-6 in mouse hepatocytes. Primary hepatocytes from wild-type (open symbols) and MCP-1^−/−^ (closed symbols) mice were cultured under normoxic (Panels **A** and **B**) and hypoxic (Panels **C** and **D**) conditions for 1, 3, 6, 24, 48 and 72 h followed by measurement of IL-6 in both lysates and supernatants using the Luminex™ xMAP technology as described in the *Materials and Methods*. Results are the mean ± SEM (n = 4–8 independent experiments, analyzed by Two-way ANOVA, *P*<0.05 (wild-type vs. MCP-1^−/−^) for all conditions except hypoxia lysates, Panel **C**).

Because one-way ANOVA and DyNA revealed that the most significant changes in inflammatory mediator production and release were observed for MCP-1 and IL-6, we hypothesized that MCP-1 would affect IL-6 expression and release in stressed hepatocytes. IL-6 levels were significantly lower in both normoxia lysates and supernatants of MCP-1^−/−^ hepatocytes as compared to normoxic (**Fig. 5A–B**) and hypoxic (**Fig. 5C–D**) wild-type controls. These results suggest that the differential expression and release of IL-6 in mouse hepatocytes depends, at least in part, on MCP-1.

### *In situ* hepatocyte expression of MCP-1 and IL-6 is attenuated in MCP-1^−/−^ cells

**Figure 6:**
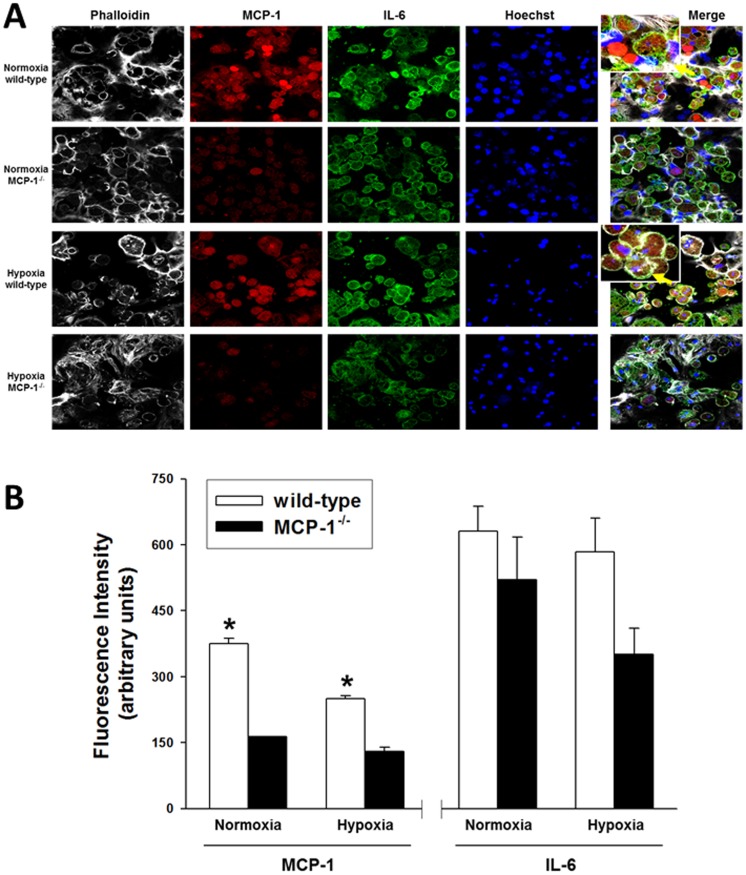
Differential expression of MCP-1 and IL-6 in wild-type and MCP-1^−/−^ hepatocytes. Primary hepatocytes isolated from wild-type and MCP-1^−/−^ mice (n = 3 each) were cultured under normoxic and hypoxic conditions for 48 h in three independent experiments. The cells were then fixed and processed for confocal immunofluorescence imaging as described in the *Materials and Methods*. (**A**) Fluorescent labeling: Phalloidin (white), MCP-1 (red), IL-6 (green), Hoechst (blue). (**B**) Quantification of immunostained cells (fluorescence intensity from 600–700 hepatocytes from 3 independent fields, n = 3 coverslips/experiment). Results are the mean ± SEM (*P<0.001 wild-type vs. MCP-1^−/−^, analyzed by *t*-test).

The differential expression of MCP-1 and IL-6 in both wild-type and MCP-1^−/−^ cells was confirmed using confocal immunofluorescence (**Fig. 6A**). Quantitative analysis of the images (**Fig. 6B**) revealed that MCP-1 is indeed elevated in wild-type hepatocytes as compared to MCP-1^−/−^ cells, with much higher levels in normoxia vs. hypoxia, confirming the results obtained by Luminex measurements (see **Fig. 1A**). Similarly, cellular IL-6 levels were lower in the MCP-1^−/−^ cells as compared to wild-type hepatocytes, especially under hypoxic conditions (**Fig. 6B**).

### Elevated plasma MCP-1 levels as biomarker for mortality and morbidity in human trauma/hemorrhage

**Figure 7:**
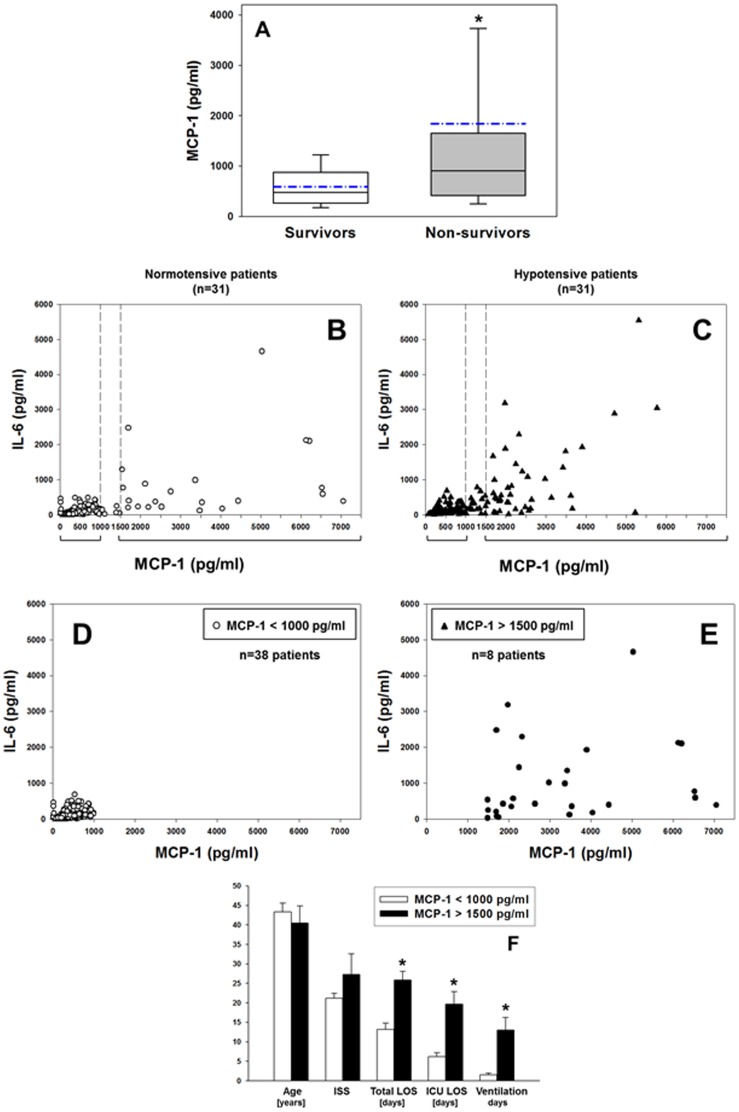
Elevated plasma MCP-1 levels are associated with mortality and morbidity in human trauma/hemorrhage. (**A**) MCP-1 levels in plasma samples from trauma patients (survivors vs. non-survivors, n = 15 each) taken within the first 24 h after trauma as described in the *Materials and Methods*. Results represent the mean ± SEM (The dotted line represents the mean value, *P = 0.001, survivors vs. non-survivors analyzed by Mann-Whitney *U* test). (**B–C**) Plot of IL-6 vs. MCP-1 levels in blood samples from normotensive (**B**) and hypotensive (**C**) trauma patients (n = 31 each) taken within the first 24 h after trauma, followed by sampling at 48, 72 and 96 h as described in the *Materials and Methods*. (**D–E**) Grouping of T/HS patients based on circulating MCP-1/CCL2 levels: Panel **D** shows MCP-1 values <1000 pg/ml (n = 38 patients) and Panel **E** shows values >1500 pg/ml (n = 8 patients). Panel **F** shows the overall demographics (Age, ISS, total LOS, ICU LOS, and ventilation days) of T/HS patients segregated according to plasma MCP-1 levels (<1000 pg/ml; [n = 38 patients] vs. >1500 pg/ml; [n = 8 patients]). Results represent the mean ± SEM. (*P<0.05, analyzed by, MCP-1<1000 pg/ml vs. MCP-1>1500 pg/ml).

Given the central role of the liver in the *in vivo* response to T/HS [17], we hypothesized that plasma MCP-1 levels would serve as an indicator of mortality in human T/HS. Indeed, an analysis of a cohort of 30 trauma patients revealed that patients could be segregated between survivors and non-survivors based on plasma MCP-1 levels within the first 24 h (**Fig. 7A**). Similar segregation could be made using plasma IL-6 levels for the same groups of patients: survivors (193.4±36.7 pg/ml) vs. non survivors (1050.1±522.9 pg/ml) (*P*<0.001, analyzed by Mann-Whitney *U* test).

MCP-1 also appeared to coordinate stress-induced production of mediators including IL-6 (see above), and IL-6 has been identified consistently as a marker of negative outcomes in T/HS [18], [19]. Accordingly, we next evaluated the potential of plasma MCP-1 levels as a biomarker for morbidity in a separate cohort of survivors of blunt trauma. A cohort of 31 hypotensive patients was matched with 31 normotensive patients (all survivors). Plotting IL-6 vs. MCP-1 plasma concentrations for the trauma patient cohort as a whole (n = 62) suggested that circulating IL-6 levels in these patients could be segregated into two groups based on circulating MCP-1 levels: low MCP-1 (<1000 pg/ml) and high MCP-1 (>1500 pg/ml, **Figs. 7B–C**). Furthermore, analysis of the data showed a positive correlation between MCP-1 and IL-6 in both normotensive (Pearson Product Moment correlation coefficient = 0.59, **Fig. 7B**) and hypotensive (0.41, **Fig. 7C**) blunt trauma patients. The *P* values associated with these correlation coefficients were highly significant (5.16×10^−17^ and 0.000047 for normotensive and hypotensive blunt trauma patients, respectively).

We then performed a different grouping irrespective of blood pressure status, based solely on MCP-1 concentrations (<1000 pg/ml vs. >1500 pg/ml). This grouping reduced the study cohort to 46 T/HS patients. T/HS patients with lower circulating MCP-1 levels (**Fig. 7D**; 400.54±15.75 pg/ml; range: 12.13–991.67 pg/ml [n = 38;]) have lower circulating IL-6 levels (106.14±8.07 pg/ml; range: 3.42–682.11 pg/ml [n = 38]) as compared to patients with higher circulating MCP-1 (**Fig. 7E**; 4098.71±772.85 pg/ml; range: 1486.46–26112.26 pg/ml [n = 8]) who exhibited higher, though more variable circulating IL-6 levels (1457.67±391.23 pg/ml; range: 26.12–10582.37 pg/ml [n = 8]).

**Figure 8:**
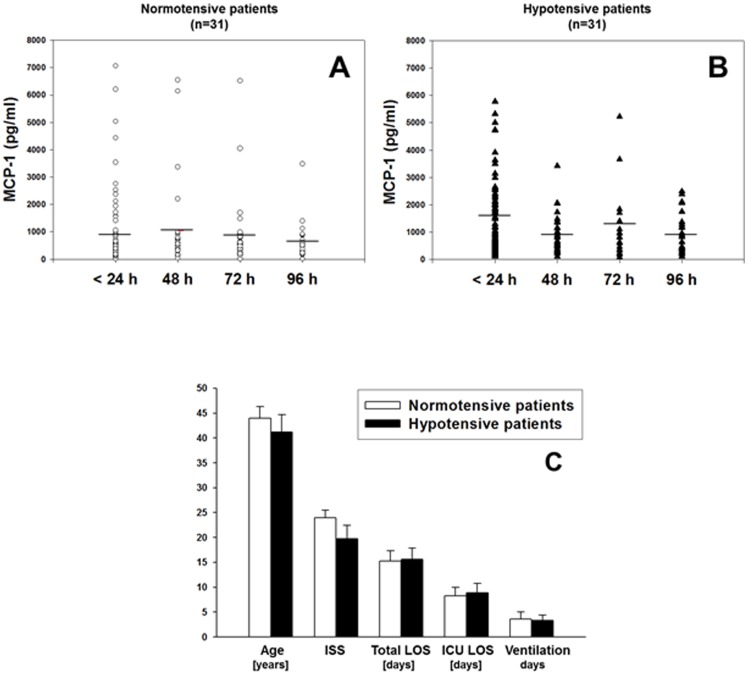
Plasma MCP-1 levels in blood samples from trauma patients. Plasma MCP-1 levels in blood samples from normotensive (Panel **A**) and hypotensive (Panel **B**) trauma patients (n = 31 each as in **Fig. 7** **Panels B–C**). Panel **C** shows the overall demographics of the normotensive (n = 27) and hypotensive patients (n = 19) segregated according to plasma MCP-1 levels (<1000 pg/ml and >1500 pg/ml) and blood pressure status. Results represent the mean ± SEM.

Patients with higher circulating MCP-1 levels had worse outcomes, as indicated by a longer total length of stay (LOS), longer intensive care unit (ICU) LOS, and greater requirement for mechanical ventilation (**Fig. 7F**). Interestingly, statistical analysis using two-way ANOVA showed no significant differences between MCP-1 levels in the initial cohort of normotensive vs. hypotensive patients (n = 31 each, **Figs. 8A–B**), and analysis of the clinical outcomes of the patients segregated based on their normotensive/hypotensive status revealed no differences between normotensive (n = 27) and hypotensive patients (n = 19) (**Fig. 8C**).

## Discussion

Liver ischemia and subsequent reperfusion, which can occur in T/HS and resuscitation, lead to inflammatory liver injury as well as to the systemic elaboration of inflammatory mediators and subsequent multiple organ dysfunction [17], [20], [21]. Liver hypoxia is a primary feature of HS in experimental models [3], [4]. Hypoxic stress, however, is but one of a variety of stresses (e.g. endoplasmic reticulum stress and autophagy) encountered by hepatocytes in settings such as T/HS [22] and I/R [23]. We therefore reasoned that the analysis of synthesis and secretion of inflammatory mediators by stressed hepatocytes would help in our understanding of the pathophysiology of T/HS and other conditions characterized, at least in part, by hepatic stress.

Our key finding is that a combined *in vitro*/*in silico* analysis of the dynamics of synthesis and secretion of multiple inflammatory mediators points to the chemokine MCP-1 as a central coordinator of the inflammatory response of stressed hepatocytes. Pursuing this *in vitro*/*in silico*-derived hypothesis allowed us to segregate blunt trauma patients based on circulating levels of MCP-1. Importantly, this segregation of patients would have proven ineffective if performed based solely on the patients' normotensive/hypotensive status post-injury.

Chemokines are small cytokine-like, heparin-binding molecules help orchestrate immune and stem cell infiltration into the liver in response to both acute and chronic injuries [20], [24]. Hepatic chemokines are released during inflammatory injury (infection, drug intoxication), and play a key role in the activation and proliferation of liver-resident cells (including hepatocytes, stellate cells, and endothelial cells). In our studies, multiple chemokines were synthesized with diverse time courses by stressed hepatocytes. Though chemokines are often thought to be highly redundant in their actions, MCP-1 was the only mediator that exhibited significant changes in both normoxia and hypoxia, and in both cell lysates (denoting cellular expression) and supernatants (denoting secretion by cells). Our findings of perturbed dynamic inflammatory networks in MCP-1^−/−^ mice supports the hypothesis that this chemokine plays a central, driving role in the hepatocyte response to stress. The apparently incomplete elimination of MCP-1 (or presence of a protein highly-homologous to MCP-1) in MCP-1^−/−^ cells raises the possibility that the actual effects of MCP-1 on coordinating inflammation might be even more pronounced than those we observed in these studies.

MCP-1 (CCL2) was first identified as a monocyte-specific chemoattractant, and was later shown to attract memory T lymphocytes and NK cells. Binding to its seven-transmembrane G-protein-coupled receptor CCR2 (CC chemokine receptor 2) results in signaling events that lead to the recruitment of monocytes in a variety of *in vivo* experimental models of infection and injury [25], [26]. In addition to recruiting monocytes, MCP-1 can also activate macrophages and endothelial cells and has been implicated in the attraction of neutrophils and the generation of neutrophil-dependent tissue damage [26]. Lu *et al.* showed that despite normal numbers of circulating leukocytes and resident macrophages, MCP-1^−/−^ mice were specifically unable to recruit monocytes 72 h after intraperitoneal thioglycollate administration, indicating that MCP-1 is essential for monocyte recruitment and the expression of cytokines related to T helper responses *in vivo* [27].

MCP-1 can be upregulated in the liver *in vivo* by I/R [28] as well as by bacterial endotoxin [29], and in isolated mouse hepatocytes by hypoxia [30] and the present study). The expression of MCP-1 in normoxic, primary cultured hepatocytes was thought to be due to the stress of cell isolation [31], and we likewise found elevated MCP-1 in normoxic hepatocytes. The effects of MCP-1 in the liver range from protective (e.g. in experimental acetaminophen-induced injury) to detrimental (e.g. during experimental I/R injury) [30].

In addition to MCP-1, our studies suggest that IL-1α, IL-6, KC, and MIG exhibit the most significant dynamic changes in stressed hepatocytes. Our results suggest that MCP-1 regulates the production of these other inflammatory mediators. This hypothesis is supported by prior studies, in which the expression of hepatic pro-inflammatory cytokines (including IL-6 and KC) was induced in alcohol-fed wild-type, but inhibited in MCP-1^−/−^ animals [32]. In support of this central role for KC, Frink *et al* showed that neutralization of this chemokine ameliorated liver damage after T/HS in mice. These authors also showed that MCP-1 causes organ damage via upregulation of KC, supporting our findings of a network of hepatic inflammation that involves these two chemokines. However, the exact source of KC remained unknown [33]; our present study suggests that hepatocytes are a key source of this chemokine.

Multiple studies have shown that IL-6 is a biomarker for adverse outcomes in settings such as trauma/hemorrhage and sepsis and specifically of poor outcome in trauma [18], [19]. This was reflected in our own clinical cohort, since we could segregate survivors from non-survivors based on plasma IL-6 levels. Importantly, our studies suggest that IL-6 is highly correlated with MCP-1 in T/HS patients, and that the production of IL-6 by hypoxic hepatocytes was essentially abolished in the absence of endogenous MCP-1. Taken together, our studies suggest that MCP-1 drives IL-6 production by hepatocytes. Interestingly, a recent study suggests the presence of a positive feedback loop involving IL-6 and MCP-1 in a setting of vascular inflammation [34].

The use of applied mathematical models and algorithms constitute a novel tool for interpreting complex biological data. In 2005, a pioneering study in this field reported on a systems model comprising a large number of intracellular signaling events directly linked to outputs associated with apoptosis, created in order to study how molecular information is processed as a network [35]. Prior studies have also utilized advanced computational analyses to discern key features of the inflammatory biology of hepatocytes. For example, Alexopoulos *et al* studied 26,000 protein state measurements from isolated primary human hepatocytes and HepG2 liver cancer cells exposed to growth factors or inflammatory mediators, yielding interaction graphs using multilinear regression [36]. The authors suggested major differences between primary and transformed hepatocytes with respect to Toll-like receptor-4 signaling and NF-κB-dependent secretion of chemokines and cytokines, and suggested, as we do here, that combined *in vitro*/*in silico* analyses can yield novel insights into hepatocyte biology [36]. More recently, Boolean logic models of immediate-early signaling in liver cells were created by training a literature-based prior knowledge network against biochemical data obtained from primary human hepatocytes as well as four hepatocellular carcinoma cell lines exposed to combinations of cytokines and small-molecule kinase inhibitors [37]. Our present study extends this paradigm to define key nodes in the stress-induced inflammatory networks induced in hepatocytes, as well as suggesting higher-order, clinically-applicable insights. Indeed, this study points to various similarities between hepatocytes *in vitro* and T/HS *in vivo*. For example, as in the present study, we found that, together with MIG, IL-6 was highly elevated but disconnected from other inflammatory mediators comprising a dynamic network of inflammation induced by experimental T/HS in mice [12]. Our present study raises the possibility that MCP-1 is the stimulus for post-HS MIG and IL-6.

In the present study, we developed a novel algorithm for consensus clustering of multiple data-driven analyses of dynamic responses. We hypothesized that those inflammatory mediators exhibiting the most coordination across experimental conditions could be important drivers or indicators of that process. The strategy we employed was to extract patterns from dynamic data, and to assess the measure of similarity across multiple dynamic patterns. To distill the most important information from three independent clustering results over 18 mediators for each of 4 experimental conditions, we filtered the results by discarding inflammatory mediators that showed inconsistent segregation patterns across the three analyses. A consensus clustering containing only the mediators whose coordination patterns in which we could be most confident was the basis of comparison across experimental conditions. This method identified MCP-1 as the most relevant mediator in our studies.

This novel approach to data-driven modeling helped formulate a key hypothesis of our study, namely that circulating levels of MCP-1 could serve as a biomarker for mortality in human trauma/hemorrhage, and that the outcomes of trauma survivors would differ in high- vs. low-producers of MCP-1. Importantly, our results suggest that circulating levels of MCP-1 are better discriminators of outcome in our moderately-injured blunt trauma survivors than hypotension. This, despite the fact that even a brief period of hypotension was suggested to elevate post-trauma morbidity and mortality in injured patients [38]. Collectively, our results provide a systems view of the hepatocyte inflammatory response to cell stress, with implications for ischemia and hemorrhage, and point to hepatocytes as a likely cellular source of cytokines/chemokines in the inflammatory response. Furthermore, these results highlight the capacity of data-driven analyses for suggesting novel, clinically-relevant targets.

## Materials and Methods

### Mouse hepatocyte isolation and culture

All procedures involving animals were approved by the Animal Care and Use Committee of the University of Pittsburgh. Hepatocytes were harvested from wild-type C57BL/6 mice (n = 8 animals from Charles River Laboratories, Wilmington, MA) or MCP-1/CCL2-null (MCP-1^−/−^) mice (n = 4 animals from The Jackson Laboratory, Bar Harbor, ME) [27] and plated as previously described [39]. After overnight incubation, the medium was removed and cells were further incubated with fresh media containing 5% heat-inactivated calf serum. Hypoxic conditions were obtained by placing the cells into a modular incubator chamber (Billups-Rothenburg, Del Mar, CA) flushed with a hypoxic gas mixture containing 1% O_2_, 5% CO_2_ and 94% N_2_. Normoxic hepatocytes served as control.

### Protein isolation, sample preparation and Western blot analysis

At the end of each experiment, the supernatants were stored at −20°C until further analysis and the cell monolayers were washed twice with ice-cold PBS and resuspended in ice-cold lysis buffer (Cell Lysis Buffer 10× from Cell Signaling Technology, Danvers, MA) containing the protease inhibitors leupeptin (0.1 µg/ml) and phenylmethylsulfonyl fluoride (1 mM) followed by total protein isolation as previously described [39]. Protein samples (50 µg) were separated on 15% SDS-polyacrylamide gels followed by electroblotting onto PVDF nitrocellulose membranes. Immunodetection of MCP-1 was done using a mouse specific rabbit polyclonal anti-MCP-1 antibody (Cell Signaling, Danvers, MA) at 1∶1000 dilution, and the immunoreactive bands were visualized after incubation with the SuperSignal West Dura Extended Duration Substrate mouse kit (Thermo Scientific, Rockford, IL). For normalization, the membranes were stripped and re-probed with an anti-β-actin antibody from Abcam (Cambridge, MA).

### Measurement of inflammatory mediators

Mouse inflammatory mediators were detected in both cell lysates and supernatants using the Milliplex™ Mouse Cytokine/Chemokine Panel I beadset (Millipore, Billerica, MA) and the Luminex™ 100 IS system (Luminex, Austin, TX) as per manufacturer's specifications. The antibody bead kit included: Granulocyte-Macrophage Colony-Stimulating Factor (**GM-CSF**), Interferon-γ (**IFN-γ**), Interleukin (**IL**)**-1α, IL-1β, IL-2, IL-4, IL-5, IL-6, IL-10, IL-13, IL-17**, Interferon-γ-inducible Protein 10 (**IP-10/CXCL10**), Keratinocyte-derived Cytokine (**KC/CXCL1**), Monocyte Chemoattractant Protein-1 (**MCP-1/CCL2**), Monokine induced by Interferon-γ (**MIG/CXCL9**), Macrophage Inflammatory Protein-1α (**MIP-1α/CCL3**), Tumor Necrosis Factor-α (**TNF-α**), and Vascular Endothelial Growth Factor (**VEGF**).

### Immunocytochemistry

Freshly isolated mouse hepatocytes (wild-type and MCP-1^−/−^) plated on coverslips (2×10^5^ cells/22-mm glass coverslip, BD Biocoat, Bedford, MA) were cultured under normoxic (control) or hypoxic (1% O_2_) conditions for 48 h. Cells were then fixed and visualized as described previously [40]. For immunostaining the following primary and secondary antibodies (diluted in PBS containing 1% donkey serum) were used: LEAF™ Purified anti-mouse MCP-1 antibody (1∶2000; Cat. No. 505905, BioLegend, San Diego, CA), rabbit polyclonal anti-IL-6 antibody (1∶2000; Cat. No. ab6672, Abcam), Cy3-conjugated anti-mouse (1∶1000; Cat. No. 715-165-151, Jackson-ImmunoResearch Laboratories, West Grove, PA), Alexa Fluor-conjugated 647 anti-rabbit (1∶1000; Cat. No. A31573, Invitrogen, Grand Island, NY), and 488-conjugated anti-phalloidin (1∶1000; Cat. No. A12379, Invitrogen). For nuclei detection the slides were counterstained with Hoechst 33342 (1∶3000) for 5 min at room temperature followed by 3 washes in PBS for 10 min.

### Statistical analysis and data-driven modeling

Before the statistical analyses and in order to account for experimental variability in cell number and protein concentration between individual experiments, the final mediator concentrations adjusted for protein content (Cf) were obtained from the Luminex analysis for each individual mediator measurement (C) and its respective lysate protein concentration (as determined by BCA, see above) using the following formulas:
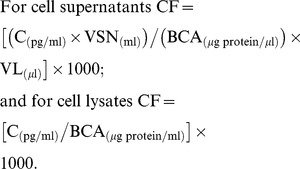
The final mediator concentrations (Cf) are thus expressed in pg/mg total protein, where the supernatant volume (Vsn) and lysis buffer volume (Vl) values used were as follows: 60 mm Petri dish (3×10^6^ cells, Vsn = 3 ml, Vl = 200 µl) and 100 mm Petri dish (5×10^6^ cells, Vsn = 5 ml, Vl = 400 µl).

Experimental data are presented as mean ± SEM. Analysis was performed by *t*- test, One-way or Two-way Analysis of Variance (ANOVA) followed by the Holm-Sidak *post-hoc* test as indicated. Significance of differences in clinical outcomes was determined by Mann-Whitney *U* test using SigmaPlot (Systat Software, San Jose, CA).

Before any dynamic analysis, the final mediator concentrations (Cf) were first normalized for each inflammatory mediator, so that all mediator levels were converted into the same scale (from 0 to 1). All measurements of a given inflammatory mediator from a single animal were normalized as a group, by dividing each value by the Euclidean norm of the group. In this way, any artifactual effects on variance due to the different ranges of concentration observed for different mediators were eliminated.

### Hierarchical clustering

Fold change values were calculated by dividing the difference between the later (x_2_) and earlier (x_1_) mediator values by the earlier value: (x_2_-x_1_)/x_1_; we avoided division by 0 by replacing x_1_ with a very small number (10^−5^) when necessary. The columns of the dendrogram contained the mean of fold changes across all animals. We employed several MatLab® functions to carry out this analysis: *linkage*, with average link and Pearson correlation distance, and *cluster* was used to find 2–4 clusters in both fold change and raw data (mediator concentrations adjusted for protein content vs. time, unaveraged, all animals included).

### Principal Component Analysis and k-means clustering

Normalized inflammatory mediator data (each measurement taken to be a single point in 18-dimensional inflammatory mediator space) were transformed into principal component space using the MatLabR function, *princomp*. We then examined projections of inflammatory mediators into principal component space by using the score coefficients for the first 2, 3, or 4 principal components. We employed k-means clustering using the MatLab® function, *kmeans*, using as a distance metric the cosine of the angle between a point and the origin. We computed clusters starting with k = 2, 3, or 4 random centroids. At each level of k, we repeated the clustering 10 times, resetting new centroids each time and taking the lowest mean distance between points and their nearest centroids as the best result.

### Meta-clustering

The Adjusted Rand Index (ARI) was first calculated pairwise between clustering results including 2, 3, or 4 principal components, choosing the result that captured the most information with the fewest principal components. We then used ARI to compare hierarchical clustering results with k = 2, 3, and 4 to k-means clustering results with k = 2, 3 and 4 in a pairwise manner. The maximum Rand Index dictated the k of the hierarchical and k-means clustering results that were moved forward into further analysis. We compared PCA k-means clustering to each of fold change hierarchical clustering and raw data hierarchical clustering individually. The k was chosen for k-means clustering that maximized both ARI values. We then filtered the results by discarding inflammatory mediators that showed inconsistent segregation patterns across the three analyses. The basis of comparison across different experiments was a consensus clustering containing only the mediators for whose coordination patterns we had confidence, as shown in detail in **Figures S9, S10, S11, S12, S13, S14, S15, S16**. Briefly, a block of data was constructed with one mediator in each row and its corresponding cluster label for each of the three results in the columns using Microsoft Excel®. Sorting this block of data by each of the cluster label columns successively allowed us to compare the memberships of clusters across the three results and to identify corresponding clusters between results. These were clusters that contained a common set of mediators across all three results. We used the following heuristic to determine consensus: once a cluster from one result was associated with clusters from the other results, (e.g. Cluster A from Raw Data was associated with Cluster 2 from Fold Change and Cluster IV from PCA clustering) it could not be associated with any other clusters. For example Cluster 1 could not associate with both Cluster A and Cluster B. Mediators that did not segregate into corresponding clusters across all results were deemed inconsistent and omitted from the consensus clustering result. This consensus outcome became the basis of comparison among experimental conditions (see **Figures S9, S10, S11, S12, S13, S14, S15, S16**).

### Dynamic Network Analysis (DyNA)

Inflammatory mediator networks were created over time periods between consecutive measurements (1–3 h, 3–6 h, 6–24 h, 24–48 h, and 48–72 h) using Matlab® and Inkscape® software (http://inkscape.org/). In order to be included in the DyNA, a given mediator had to be statistically significantly different from its baseline value (no treatment [time = 1 h]; *P*<0.05 by Student's t-test). Connections in the network were created if the correlation coefficient between two nodes (inflammatory mediators) was greater or equal to a threshold of 0.7 (based on a total of 12 samples with 10 degrees of freedom, *P*<0.05).

### Human trauma patient selection and analysis

All human sampling was done following approval by the University of Pittsburgh Institutional Review Board and written informed consent was obtained from each patient or next of kin as per Institutional Review Board regulations. From a cohort of 493 blunt trauma patients admitted to Presbyterian University Hospital (part of the University of Pittsburgh Medical Center), we selected two cohorts of blunt trauma patients (all ≥18 years old). Exclusion criteria included: life expectancy <24 h, penetrating trauma only, and traumatic brain injury. The first cohort included 30 trauma patients separated into two groups: 15 survivors (mean age: 60.5±3.7 years, range: 43–86 years, mean ISS: 24.5±2.8, range: 9–50), and 15 non-survivors (mean age: 59.5±4.9 years, range: 25–86 years, mean ISS: 24.8±2.3, range: 10–38). The second cohort included 62 patients (all survivors) separated into two groups matched on age, gender, and Injury Severity Score (ISS): 31 normotensive patients (19 males and 12 females; systolic blood pressure >90 mmHg, mean age: 44.4±2.2 years, range: 24–75 years, mean ISS: 23.5±1.3, range: 10–38), matched with 31 hypotensive patients (19 males and 12 females; systolic blood pressure <90 mmHg, duration of hypotension post-injury: 8±1 h, range: 2–21 h, mean age: 45.1±3.4 years, range: 20–89 years, mean ISS: 23.5±2.2, range: 4–54). One to four blood plasma samples per patient were collected within the first 24 h post-injury, followed by additional sampling at 48, 72, and 96 h. All blood samples were stored at −80°C until analysis. MCP-1 and IL-6 were measured using the Luminex™ 100 IS system and the Human 25-plex® Luminex™ beadset (BioSource, now Invitrogen) or the Milliplex™ MAP kit (Millipore, Billerica, MA).

## Supporting Information

Figure S1 **Relevant inflammatory mediator groupings in the hepatocyte response to normoxia as determined by consensus of clustering methods.** Primary hepatocytes from **wild-type** mice were cultured under normoxic (control, 21% O_2_) conditions for 1, 3, 6, 24, 48 and 72 h as described in the *Materials and Methods*. At the end of the experiments, samples from **lysates** (L) and supernatants (SN, see Fig. **S3** below) were assayed for 18 mouse inflammatory mediators using the Luminex xMAP technology, the measurements were normalized for protein content and over each inflammatory mediator and each sample and k-means clustering was performed over dynamic inflammatory mediator measurements from 4 experimental conditions (N vs. H, and L vs. SN) using MatLab® software as described in the *Materials and Methods*. The tridimensional grouping of significant inflammatory mediators according to k-means clustering is represented and the percentage of total variance corresponding to the analysis is shown on the top of the graph.
(TIF)

Figure S2 **Relevant inflammatory mediator groupings in the hepatocyte response to hypoxia as determined by consensus of clustering methods.** Primary hepatocytes from **wild-type** mice were cultured under hypoxic (1% O_2_) conditions for 1, 3, 6, 24, 48 and 72 h as described in the *Materials and Methods*. At the end of the experiments, samples from both **lysates** (L) and supernatants (SN, see Fig. **S4** below) were assayed for 18 mouse inflammatory mediators using the Luminex xMAP technology, the measurements were normalized for protein content and over each inflammatory mediator and each sample and k-means clustering was performed over dynamic inflammatory mediator measurements from 4 experimental conditions (N vs. H, and L vs. SN) using MatLab® software as described in the *Materials and Methods*. The tridimensional grouping of significant inflammatory mediators according to k-means clustering is represented and the percentage of total variance corresponding to the analysis is shown on the top of the graph.
(TIF)

Figure S3 **Relevant inflammatory mediator groupings in the hepatocyte response to normoxia as determined by consensus of clustering methods: wild-type supernatant.** (See legend of Figure **S1** for detailed description).
(TIF)

Figure S4 **Relevant inflammatory mediator groupings in the hepatocyte response to hypoxia as determined by consensus of clustering methods: wild-type supernatant.** (See legend of Figure **S2** for detailed description)
(TIF)

Figure S5 **Relevant inflammatory mediator groupings in the hepatocyte response to normoxia as determined by consensus of clustering methods.** Primary hepatocytes from **MCP-1^−/−^** mice were cultured under normoxic (control, 21% O_2_) conditions for 1, 3, 6, 24, 48 and 72 h as described in the *Materials and Methods*. At the end of the experiments, samples from both **lysates** (L) and supernatants (SN, see Fig. **S7** below) were assayed for 18 mouse inflammatory mediators using the Luminex xMAP technology, the measurements were normalized for protein content and over each inflammatory mediator and each sample and k-means clustering was performed over dynamic inflammatory mediator measurements from 4 experimental conditions (N vs. H, and L vs. SN) using MatLab® software as described in the *Materials and Methods*. The tridimensional grouping of significant inflammatory mediators according to k-means clustering is represented and the percentage of total variance corresponding to each analysis is shown on the top of the graph.
(TIF)

Figure S6 **Relevant inflammatory mediator groupings in the hepatocyte response to hypoxia as determined by consensus of clustering methods.** Primary hepatocytes from **MCP-1^−/−^** mice were cultured under hypoxic (1% O_2_) conditions for 1, 3, 6, 24, 48 and 72 h as described in the *Materials and Methods*. At the end of the experiments, samples from both **lysates** (L) and supernatants (SN, see Fig. **S8** below) were assayed for 18 mouse inflammatory mediators using the Luminex xMAP technology, the measurements were normalized for protein content and over each inflammatory mediator and each sample and k-means clustering was performed over dynamic inflammatory mediator measurements from 4 experimental conditions (N vs. H, and L vs. SN) using MatLab® software as described in the *Materials and Methods*. The tridimensional grouping of significant inflammatory mediators according to k-means clustering is represented and the percentage of total variance corresponding to each analysis is shown on the top of the graph.
(TIF)

Figure S7 **Relevant inflammatory mediator groupings in the hepatocyte response to normoxia as determined by consensus of clustering methods: MCP-1^−/−^ supernatant.** (See legend of Figure **S5** for detailed description).
(TIF)

Figure S8 **Relevant inflammatory mediator groupings in the hepatocyte response to hypoxia as determined by consensus of clustering methods: MCP-1^−/−^ supernatant.** (See legend of Figure **S6** for detailed description).
(TIF)

Figure S9 **Meta-clustering process: from three independent clustering results to a consensus clustering.** Each method is colored one hue (blue = hierarchical clustering of raw data, orange = hierarchical clustering on fold changes, green = k-means clustering in PCA space). Clusters within each method are demarcated by shades and tones of each hue (**Step 1**). Each column was sorted alphabetically by mediator name (**Step 2**), and the clustering results were combined into a single matrix (**Step 3**). The rows of this matrix were sorted by cluster labels, for each method sequentially (**Step 4**). This sorting allowed visual identification of clusters that were associated across methods. From here, a consensus (**Step 6**) was determined by identifying associated clusters and removing inconsistent mediators (**Step 5**) that fell outside of the associated clusters. Associated clusters are those that contain the same mediators across all three clustering methods. If two or more clusters from one method were associated with one cluster from each of the other methods, they were considered to be part of the same cluster, provided neither cluster was associated with any other clusters. Analysis for **normoxia supernatants (wild-type):** In Step 4 for this matrix, the rows were first sorted according to fold change cluster labels (orange), then by raw data cluster labels (blue), and finally by PCA clusters (green). In Step 5, all mediators from Cluster A (light blue) were also found in Cluster 2 (bright orange) and Cluster IV (dark green). Likewise, all mediators from Cluster 1 (light orange) were found in Clusters B (bright blue) and II (light green). Members of those clusters that were not found in Cluster 1 were discarded.
(TIF)

Figure S10 **Meta-clustering analysis (see legend of Fig. S9) for hypoxia supernatants (wild-type): In Step 4 for this matrix, the rows were first sorted according to raw data cluster labels (blue), then by fold change cluster labels (orange), and finally by PCA clusters (green).** In Step 5, all mediators from Cluster D (dark blue) were also found in Cluster 2 (bright orange) and Cluster II (light green) and therefore, these three clusters were considered to be the same. Any mediators associated with Cluster II, but not with both Cluster 2 and Cluster D were then marked as inconsistent and discarded from the analysis. Clusters I (grey), III (medium green), and IV (dark green) were all associated with Cluster B (bright blue) and Cluster 2 (bright orange).
(TIF)

Figure S11 **Meta-clustering analysis (see legend of Fig. S9) for normoxia lysates (wild-type): In Step 4 for this matrix, the rows were first sorted according to fold change cluster labels (orange), then by raw data cluster labels (blue), and finally by PCA clusters (green).** In Step 5, all mediators from Cluster C (medium blue) were also found in Cluster 1 (light orange) and Cluster I (grey) and therefore, these three clusters were considered to be the same. Any mediators associated with Cluster C, but not with both Cluster 1 and Cluster I were then marked as inconsistent and discarded from the analysis. Clusters B (bright blue) and D (dark blue) were both associated with Cluster II (light green) and Cluster 2 (bright orange).
(TIF)

Figure S12 **Meta-clustering analysis (see legend of Fig. S9) for hypoxia lysates (wild-type): In Step 4 for this matrix, the rows were first sorted according to fold change cluster labels (orange), then by raw data cluster labels (blue), and finally by PCA clusters (green).** In Step 5, most mediators from Cluster A (light blue) were also found in Cluster 2 (medium orange) and Cluster I (grey) and therefore, these three clusters were considered to be the same. Any mediators associated with Cluster A, but not with both Cluster 2 and Cluster I were then marked as inconsistent and discarded from the analysis. Clusters C (medium blue) and D (dark blue) were both associated with Cluster 4 (dark orange) and Cluster III (medium green).
(TIF)

Figure S13 **Meta-clustering analysis (see legend of Fig. S9) for normoxia supernatants (MCP-1^−/−^): In Step 4 for this matrix, the rows were first sorted according to fold change cluster labels (orange), then by raw data cluster labels (blue), and finally by PCA clusters (green).** In Step 5, a majority of mediators from Cluster II (light green) were also found in Cluster B (bright blue) and Cluster 1 (light orange) and therefore, these three clusters were considered to be the same. Any mediators associated with Cluster II, but not with both Cluster B and Cluster 1 were marked as inconsistent and discarded from the analysis. Likewise, Clusters D (dark blue), 2 (bright orange), and III (medium green) were associated.
(TIF)

Figure S14 **Meta-clustering analysis (see legend of Fig. S9) for hypoxia supernatants (MCP-1^−/−^): In Step 4 for this matrix, the rows were first sorted according to fold change cluster labels (orange), then by raw data cluster labels (blue), and finally by PCA clusters (green).** In Step 5, all mediators from Cluster A (light blue) were also found in Cluster 2 (bright orange) and Cluster III (medium green) and therefore, these three clusters were considered to be the same. Any mediators associated with Cluster A, but not with both Cluster 2 and Cluster III were marked as inconsistent and discarded from the analysis.
(TIF)

Figure S15 **Meta-clustering analysis (see legend of Fig. S9) for normoxia lysates (MCP-1^−/−^): In Step 4 for this matrix, the rows were first sorted according to fold change cluster labels (orange), then by raw data cluster labels (blue), and finally by PCA clusters (green).** In Step 5, all mediators from Cluster 1 (light orange) were also found in Cluster B (bright blue) and Cluster II (light green) and therefore, these three clusters were considered to be the same. Any mediators associated with Cluster 1, but not with both Cluster B and Cluster II were marked as inconsistent and discarded from the analysis. Clusters III (medium green) and IV (dark green) were both associated with Cluster B (bright blue) and Cluster 2 (bright orange).
(TIF)

Figure S16 **Meta-clustering analysis (see legend of Fig. S9) for hypoxia lysates (MCP-1^−/−^): In Step 4 for this matrix, the rows were first sorted according to raw data cluster labels (blue), then by fold change cluster labels (orange), and finally by PCA clusters (green).** In Step 5, all mediators from Cluster B (medium blue) were also found in Cluster 3 (bright orange) and Cluster III (medium green) and therefore, these three clusters were considered to be the same. Any mediators associated with Cluster B, but not with both Cluster 3 and Cluster III were marked as inconsistent and discarded from the analysis. Clusters I (grey), II (light green), and IV (dark green) were all associated with Cluster A (light blue) and Cluster 1 (light orange).
(TIF)

Figure S17 **Dynamic Network Analysis (DyNA) of inflammatory mediators produced by normoxic and hypoxic mouse hepatocytes.** Primary hepatocytes from **wild-type** mice were cultured under normoxic or hypoxic conditions (1–72 h) followed by measurement of cytokines/chemokines in both lysates and supernatants and lysates as described in the *Materials and Methods*. Gray boxes indicate that the mediator is statistically significantly different from its baseline value (no treatment [time = 1 h]; *P*<0.05) and the digits represent the number of connections resulting from the DyNAs during each of the following five time frames: 1–3 h, 3–6 h, 6–24 h, 24–48 h, and 48–72 h for both lysates and supernatants.
(TIF)

Figure S18 **Dynamic Network Analysis (DyNA) of inflammatory mediators produced by normoxic and hypoxic mouse hepatocytes from MCP-1^−/−^ mice.** Primary hepatocytes from **MCP-1^−/−^** mice were cultured under normoxic or hypoxic conditions (1–72 h) followed by measurement of cytokines/chemokines in both lysates and supernatants and lysates as described in the *Materials and Methods*. Gray boxes indicate that the mediator is statistically significantly different from its baseline value (no treatment [time = 1 h]; *P*<0.05) and the digits represent the number of connections resulting from the DyNAs during each of the following five time frames: 1–3 h, 3–6 h, 6–24 h, 24–48 h, and 48–72 h for both lysates and supernatants.
(TIF)
