# Supplementary figures and images for: Central Role for MCP-1/CCL2 in Injury-Induced Inflammation Revealed by In Vitro, In Silico, and Clinical Studies
Source: PLoS One. 2013 Dec 3;8(12):e79804. doi: 10.1371/journal.pone.0079804 (PMC3849193; doi:10.1371/journal.pone.0079804)

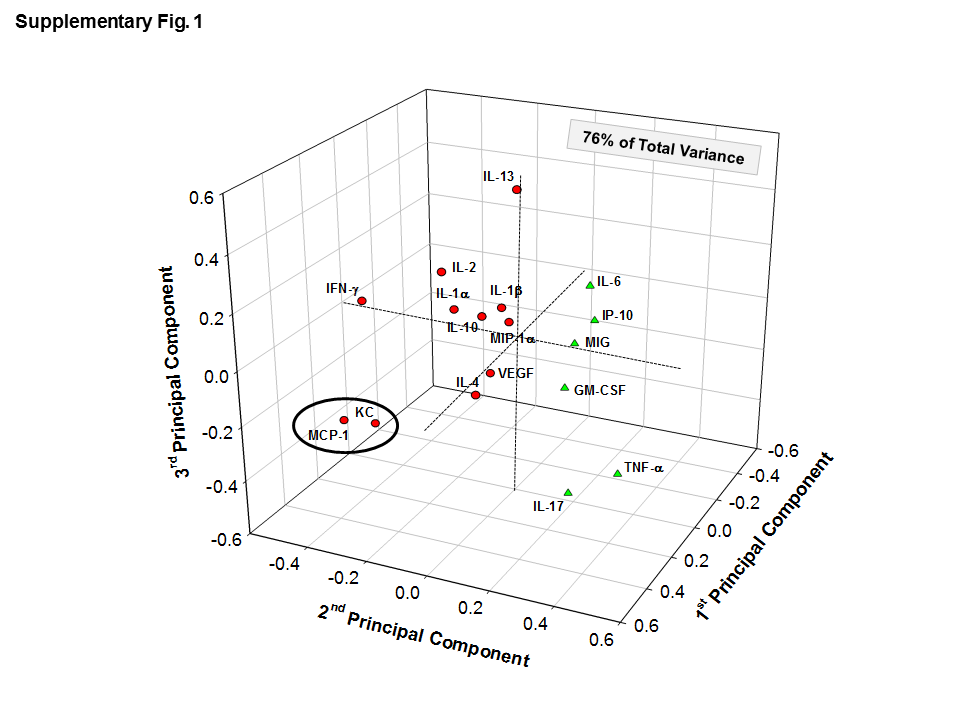

Supplement: Figure S1 — Relevant inflammatory mediator groupings in the hepatocyte response to normoxia as determined by consensus of clustering methods. Primary hepatocytes from wild-type mice were cultured under normoxic (control, 21% O2) conditions for 1, 3, 6, 24, 48 and 72 h as described in the Materials and Methods . At the end of the experiments, samples from lysates (L) and supernatants (SN, see Fig. S3 below) were assayed for 18 mouse inflammatory mediators using the Luminex xMAP technology, the measurements were normalized for protein content and over each inflammatory mediator and each sample and k-means clustering was performed over dynamic inflammatory mediator measurements from 4 experimental conditions (N vs. H, and L vs. SN) using MatLab® software as described in the Materials and Methods . The tridimensional grouping of significant inflammatory mediators according to k-means clustering is represented and the percentage of total variance corresponding to the analysis is shown on the top of the graph. (TIF) [file pone.0079804.s001.tif]

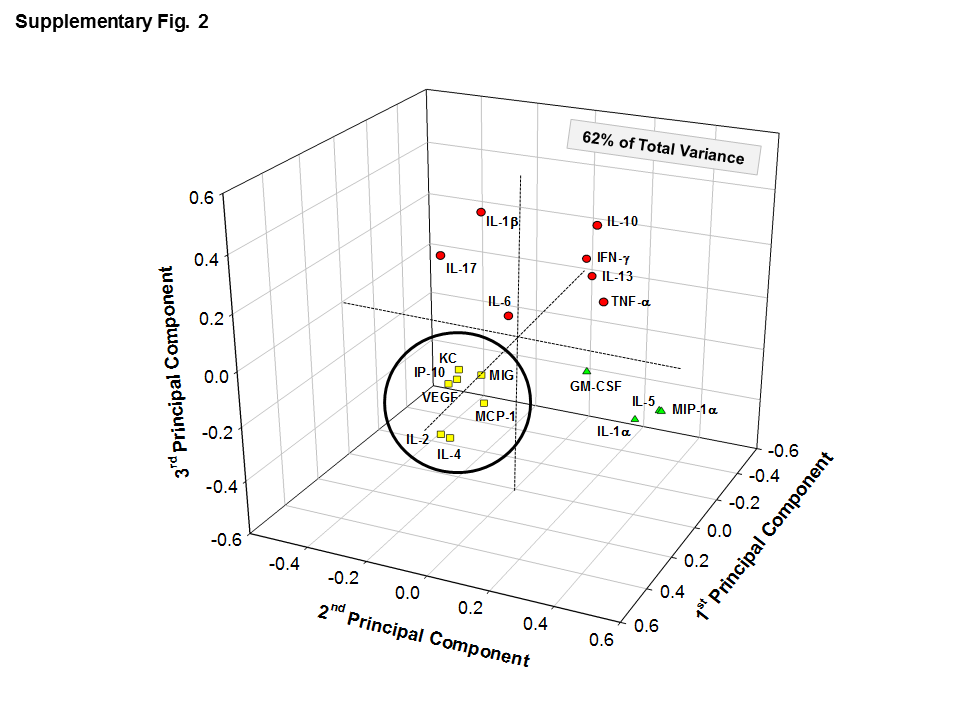

Supplement: Figure S2 — Relevant inflammatory mediator groupings in the hepatocyte response to hypoxia as determined by consensus of clustering methods. Primary hepatocytes from wild-type mice were cultured under hypoxic (1% O2) conditions for 1, 3, 6, 24, 48 and 72 h as described in the Materials and Methods . At the end of the experiments, samples from both lysates (L) and supernatants (SN, see Fig. S4 below) were assayed for 18 mouse inflammatory mediators using the Luminex xMAP technology, the measurements were normalized for protein content and over each inflammatory mediator and each sample and k-means clustering was performed over dynamic inflammatory mediator measurements from 4 experimental conditions (N vs. H, and L vs. SN) using MatLab® software as described in the Materials and Methods . The tridimensional grouping of significant inflammatory mediators according to k-means clustering is represented and the percentage of total variance corresponding to the analysis is shown on the top of the graph. (TIF) [file pone.0079804.s002.tif]

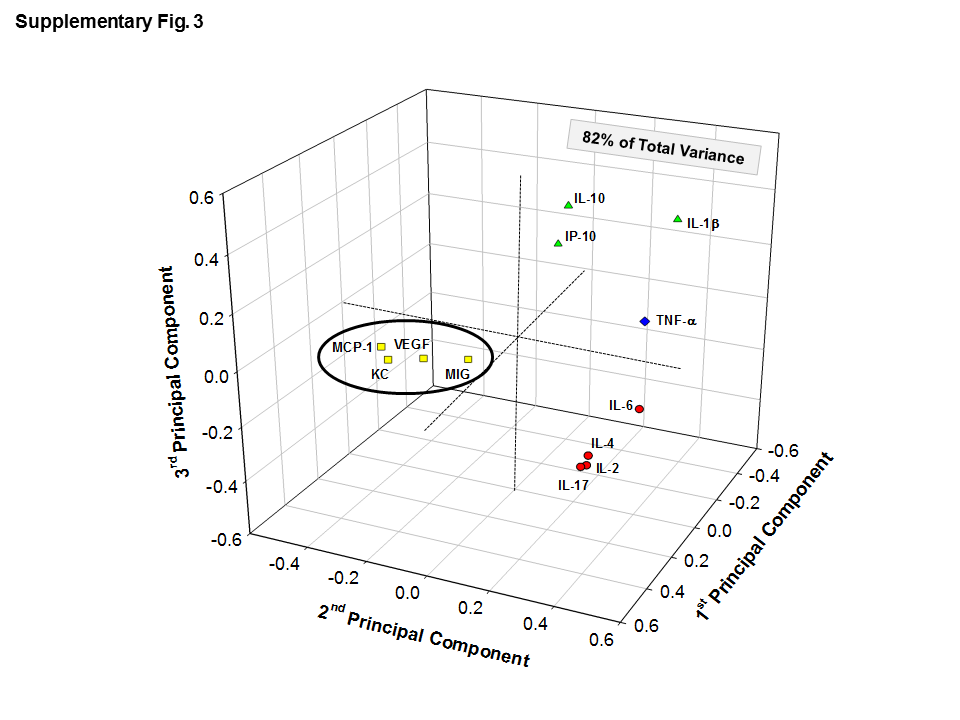

Supplement: Figure S3 — Relevant inflammatory mediator groupings in the hepatocyte response to normoxia as determined by consensus of clustering methods: wild-type supernatant. (See legend of Figure S1 for detailed description). (TIF) [file pone.0079804.s003.tif]

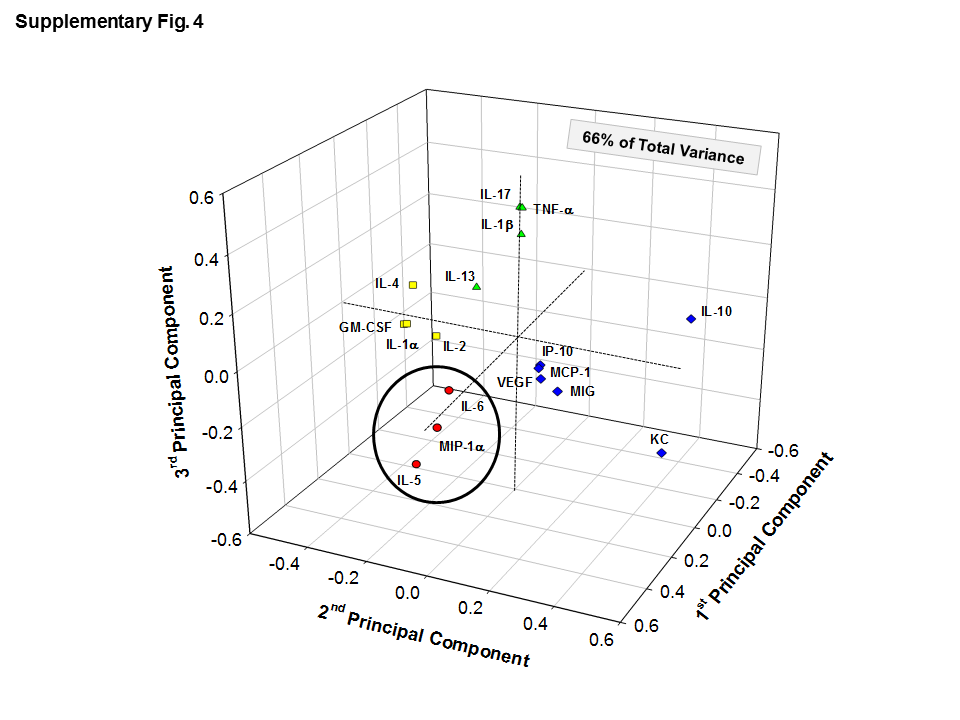

Supplement: Figure S4 — Relevant inflammatory mediator groupings in the hepatocyte response to hypoxia as determined by consensus of clustering methods: wild-type supernatant. (See legend of Figure S2 for detailed description) (TIF) [file pone.0079804.s004.tif]

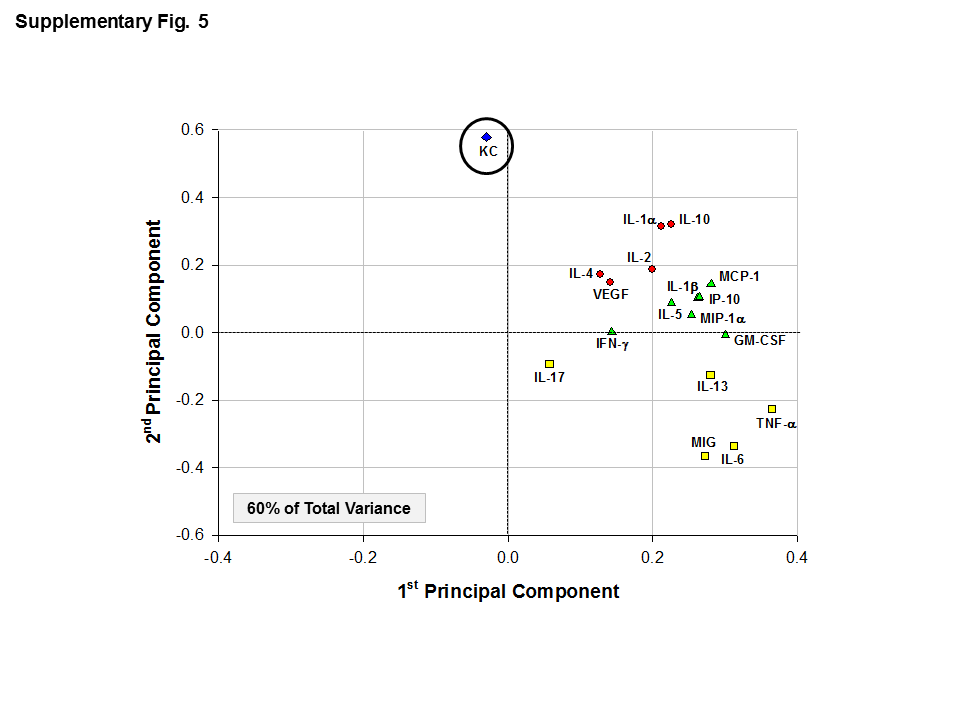

Supplement: Figure S5 — Relevant inflammatory mediator groupings in the hepatocyte response to normoxia as determined by consensus of clustering methods. Primary hepatocytes from MCP-1−/− mice were cultured under normoxic (control, 21% O2) conditions for 1, 3, 6, 24, 48 and 72 h as described in the Materials and Methods . At the end of the experiments, samples from both lysates (L) and supernatants (SN, see Fig. S7 below) were assayed for 18 mouse inflammatory mediators using the Luminex xMAP technology, the measurements were normalized for protein content and over each inflammatory mediator and each sample and k-means clustering was performed over dynamic inflammatory mediator measurements from 4 experimental conditions (N vs. H, and L vs. SN) using MatLab® software as described in the Materials and Methods . The tridimensional grouping of significant inflammatory mediators according to k-means clustering is represented and the percentage of total variance corresponding to each analysis is shown on the top of the graph. (TIF) [file pone.0079804.s005.tif]

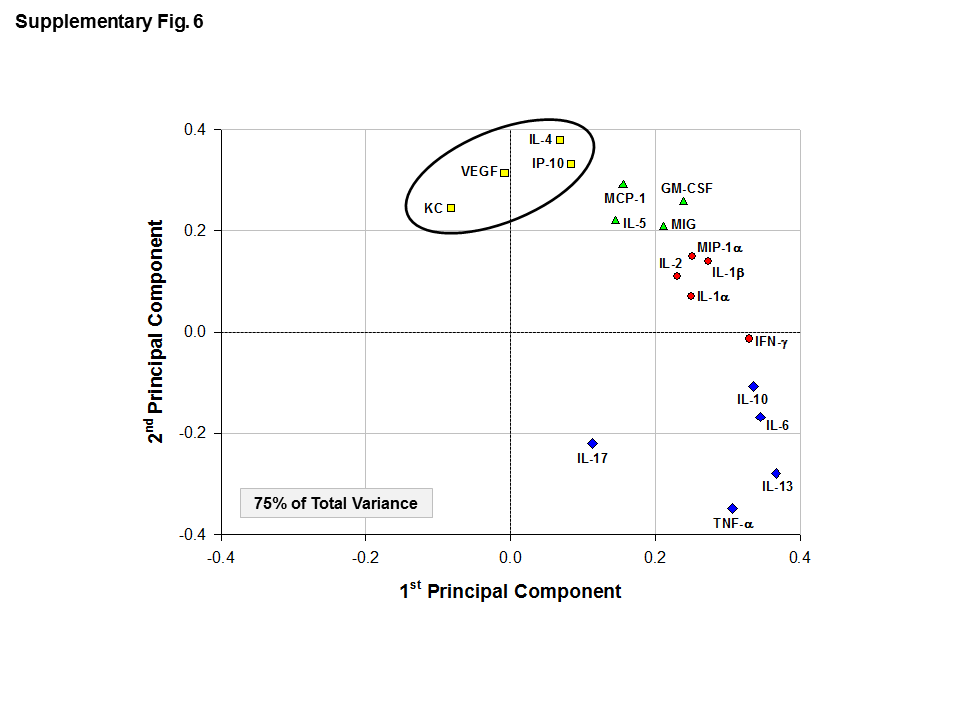

Supplement: Figure S6 — Relevant inflammatory mediator groupings in the hepatocyte response to hypoxia as determined by consensus of clustering methods. Primary hepatocytes from MCP-1−/− mice were cultured under hypoxic (1% O2) conditions for 1, 3, 6, 24, 48 and 72 h as described in the Materials and Methods . At the end of the experiments, samples from both lysates (L) and supernatants (SN, see Fig. S8 below) were assayed for 18 mouse inflammatory mediators using the Luminex xMAP technology, the measurements were normalized for protein content and over each inflammatory mediator and each sample and k-means clustering was performed over dynamic inflammatory mediator measurements from 4 experimental conditions (N vs. H, and L vs. SN) using MatLab® software as described in the Materials and Methods . The tridimensional grouping of significant inflammatory mediators according to k-means clustering is represented and the percentage of total variance corresponding to each analysis is shown on the top of the graph. (TIF) [file pone.0079804.s006.tif]

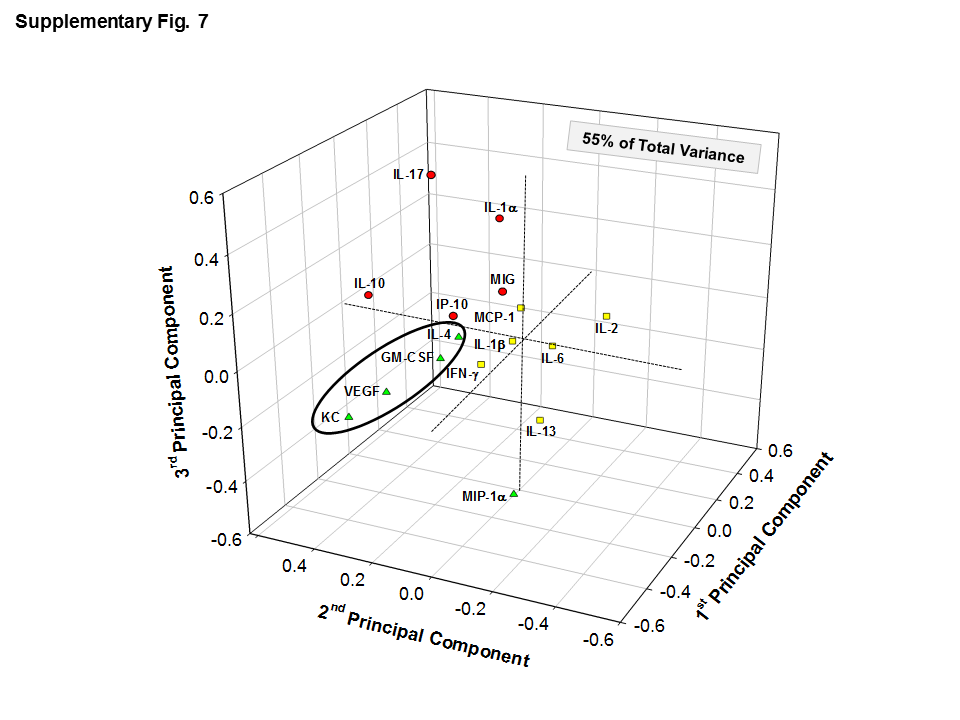

Supplement: Figure S7 — Relevant inflammatory mediator groupings in the hepatocyte response to normoxia as determined by consensus of clustering methods: MCP-1−/− supernatant. (See legend of Figure S5 for detailed description). (TIF) [file pone.0079804.s007.tif]

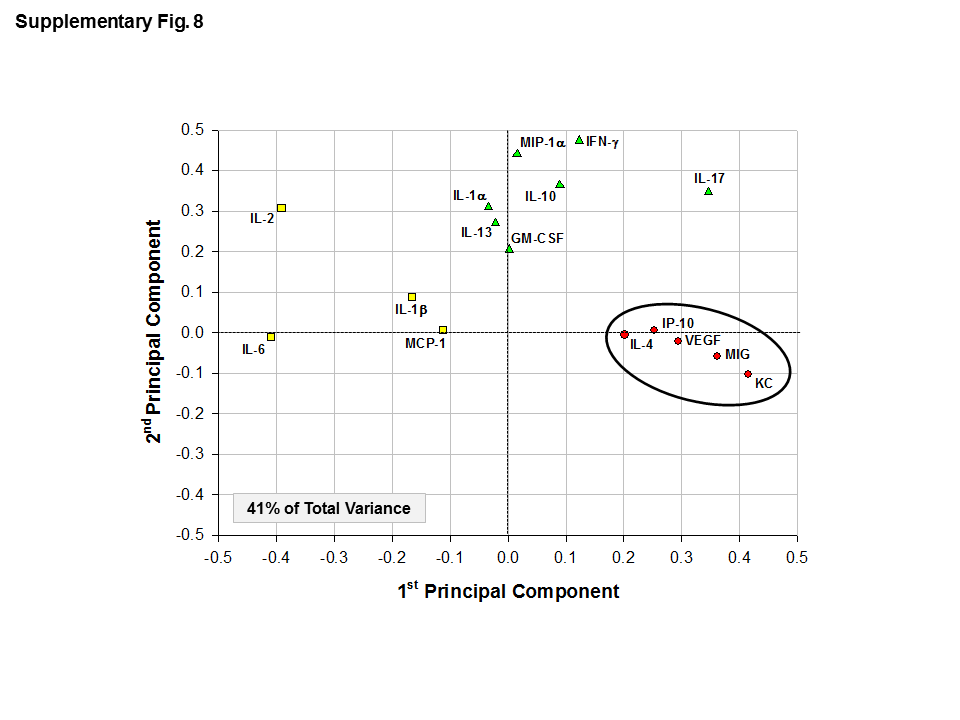

Supplement: Figure S8 — Relevant inflammatory mediator groupings in the hepatocyte response to hypoxia as determined by consensus of clustering methods: MCP-1−/− supernatant. (See legend of Figure S6 for detailed description). (TIF) [file pone.0079804.s008.tif]

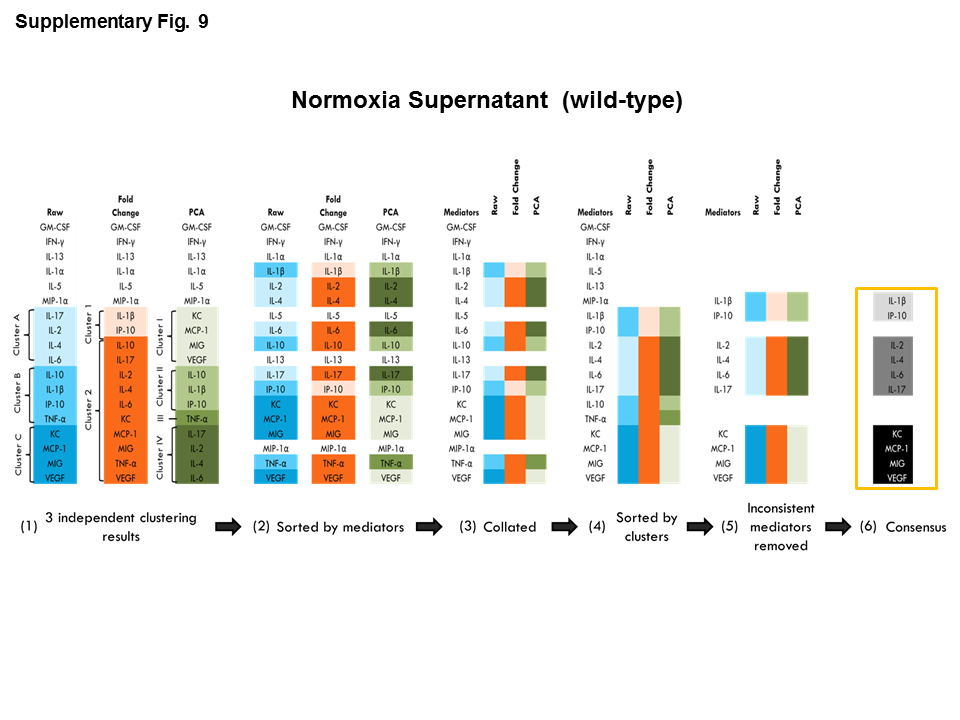

Supplement: Figure S9 — Meta-clustering process: from three independent clustering results to a consensus clustering. Each method is colored one hue (blue = hierarchical clustering of raw data, orange = hierarchical clustering on fold changes, green = k-means clustering in PCA space). Clusters within each method are demarcated by shades and tones of each hue (Step 1). Each column was sorted alphabetically by mediator name (Step 2), and the clustering results were combined into a single matrix (Step 3). The rows of this matrix were sorted by cluster labels, for each method sequentially (Step 4). This sorting allowed visual identification of clusters that were associated across methods. From here, a consensus (Step 6) was determined by identifying associated clusters and removing inconsistent mediators (Step 5) that fell outside of the associated clusters. Associated clusters are those that contain the same mediators across all three clustering methods. If two or more clusters from one method were associated with one cluster from each of the other methods, they were considered to be part of the same cluster, provided neither cluster was associated with any other clusters. Analysis for normoxia supernatants (wild-type): In Step 4 for this matrix, the rows were first sorted according to fold change cluster labels (orange), then by raw data cluster labels (blue), and finally by PCA clusters (green). In Step 5, all mediators from Cluster A (light blue) were also found in Cluster 2 (bright orange) and Cluster IV (dark green). Likewise, all mediators from Cluster 1 (light orange) were found in Clusters B (bright blue) and II (light green). Members of those clusters that were not found in Cluster 1 were discarded. (TIF) [file pone.0079804.s009.tif]

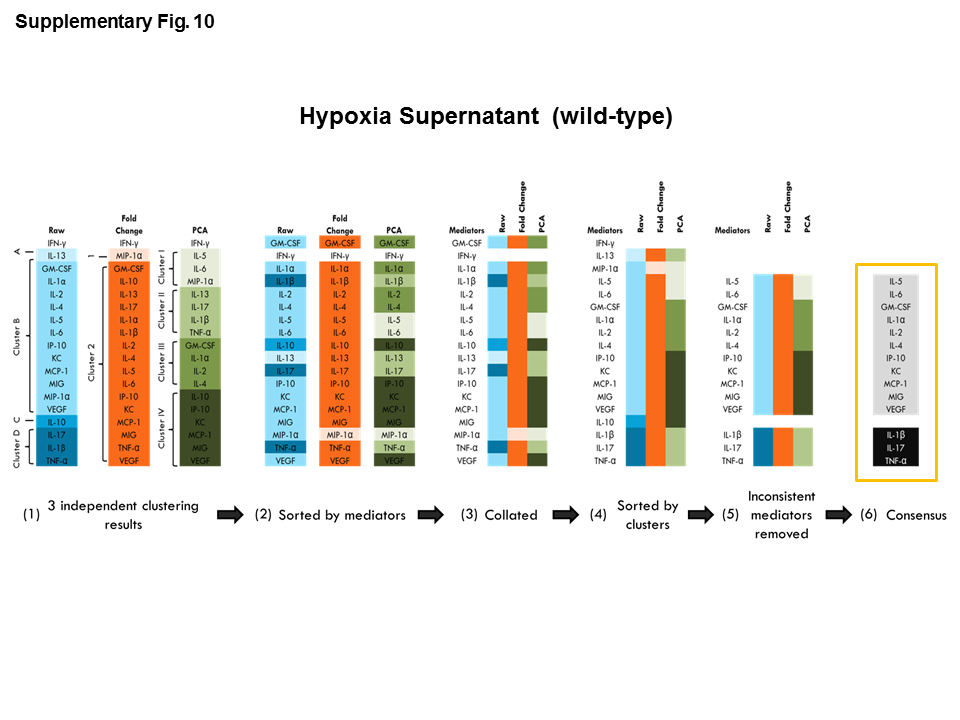

Supplement: Figure S10 — Meta-clustering analysis (see legend of Fig. S9) for hypoxia supernatants (wild-type): In Step 4 for this matrix, the rows were first sorted according to raw data cluster labels (blue), then by fold change cluster labels (orange), and finally by PCA clusters (green). In Step 5, all mediators from Cluster D (dark blue) were also found in Cluster 2 (bright orange) and Cluster II (light green) and therefore, these three clusters were considered to be the same. Any mediators associated with Cluster II, but not with both Cluster 2 and Cluster D were then marked as inconsistent and discarded from the analysis. Clusters I (grey), III (medium green), and IV (dark green) were all associated with Cluster B (bright blue) and Cluster 2 (bright orange). (TIF) [file pone.0079804.s010.tif]

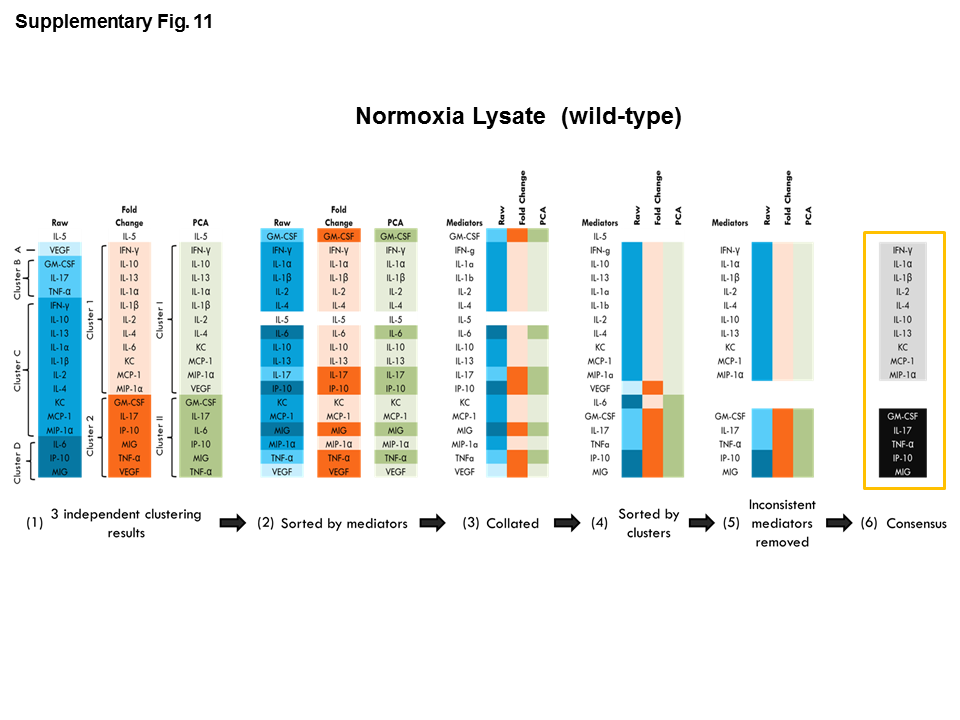

Supplement: Figure S11 — Meta-clustering analysis (see legend of Fig. S9) for normoxia lysates (wild-type): In Step 4 for this matrix, the rows were first sorted according to fold change cluster labels (orange), then by raw data cluster labels (blue), and finally by PCA clusters (green). In Step 5, all mediators from Cluster C (medium blue) were also found in Cluster 1 (light orange) and Cluster I (grey) and therefore, these three clusters were considered to be the same. Any mediators associated with Cluster C, but not with both Cluster 1 and Cluster I were then marked as inconsistent and discarded from the analysis. Clusters B (bright blue) and D (dark blue) were both associated with Cluster II (light green) and Cluster 2 (bright orange). (TIF) [file pone.0079804.s011.tif]

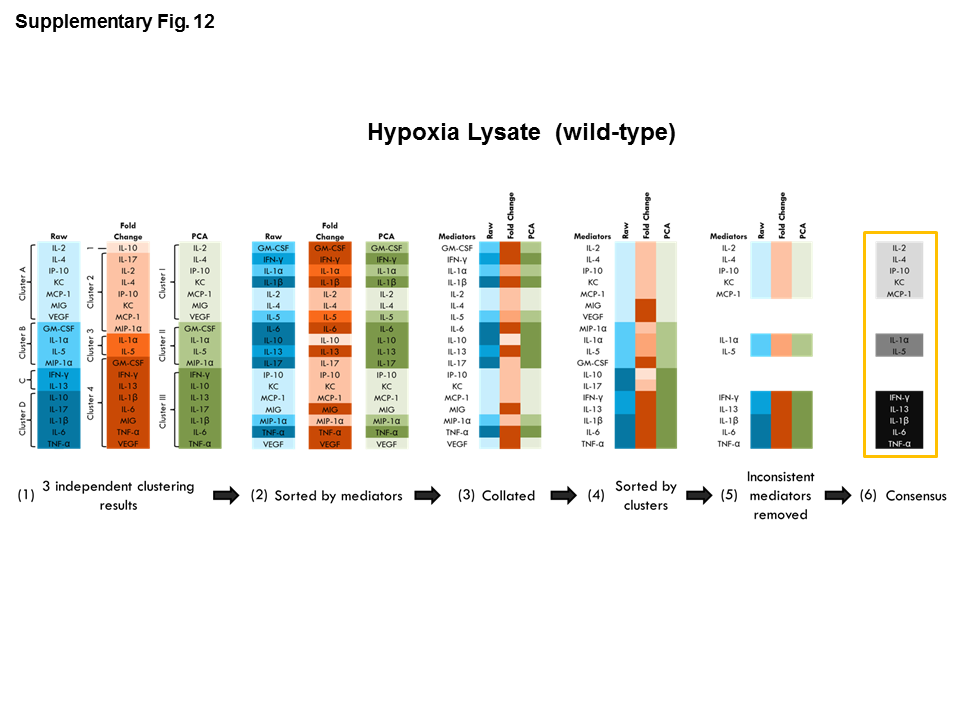

Supplement: Figure S12 — Meta-clustering analysis (see legend of Fig. S9) for hypoxia lysates (wild-type): In Step 4 for this matrix, the rows were first sorted according to fold change cluster labels (orange), then by raw data cluster labels (blue), and finally by PCA clusters (green). In Step 5, most mediators from Cluster A (light blue) were also found in Cluster 2 (medium orange) and Cluster I (grey) and therefore, these three clusters were considered to be the same. Any mediators associated with Cluster A, but not with both Cluster 2 and Cluster I were then marked as inconsistent and discarded from the analysis. Clusters C (medium blue) and D (dark blue) were both associated with Cluster 4 (dark orange) and Cluster III (medium green). (TIF) [file pone.0079804.s012.tif]

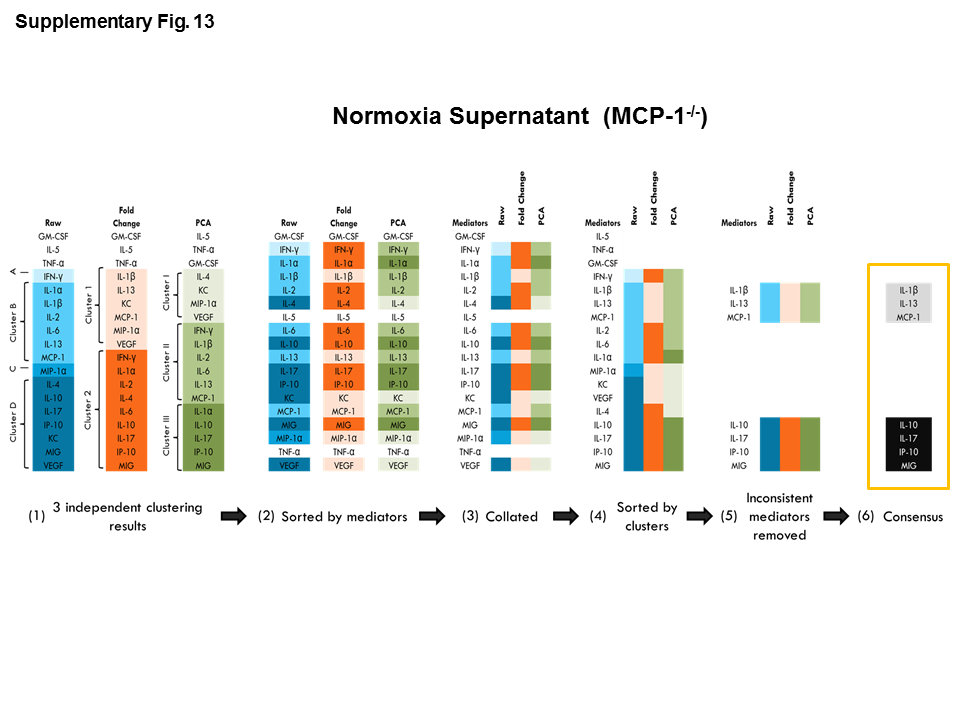

Supplement: Figure S13 — Meta-clustering analysis (see legend of Fig. S9) for normoxia supernatants (MCP-1−/−): In Step 4 for this matrix, the rows were first sorted according to fold change cluster labels (orange), then by raw data cluster labels (blue), and finally by PCA clusters (green). In Step 5, a majority of mediators from Cluster II (light green) were also found in Cluster B (bright blue) and Cluster 1 (light orange) and therefore, these three clusters were considered to be the same. Any mediators associated with Cluster II, but not with both Cluster B and Cluster 1 were marked as inconsistent and discarded from the analysis. Likewise, Clusters D (dark blue), 2 (bright orange), and III (medium green) were associated. (TIF) [file pone.0079804.s013.tif]

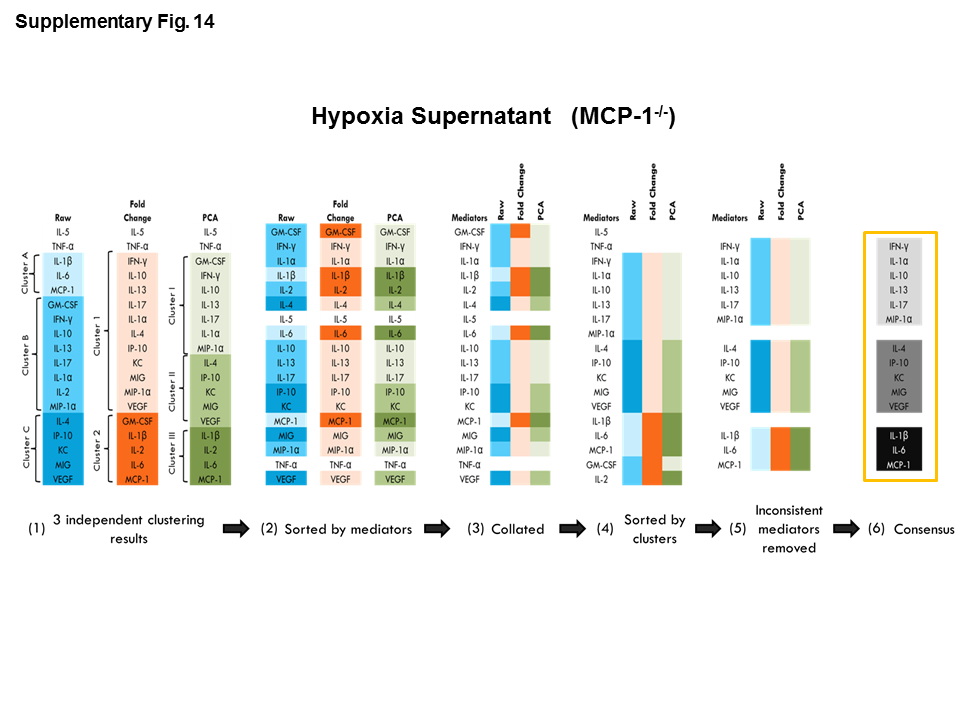

Supplement: Figure S14 — Meta-clustering analysis (see legend of Fig. S9) for hypoxia supernatants (MCP-1−/−): In Step 4 for this matrix, the rows were first sorted according to fold change cluster labels (orange), then by raw data cluster labels (blue), and finally by PCA clusters (green). In Step 5, all mediators from Cluster A (light blue) were also found in Cluster 2 (bright orange) and Cluster III (medium green) and therefore, these three clusters were considered to be the same. Any mediators associated with Cluster A, but not with both Cluster 2 and Cluster III were marked as inconsistent and discarded from the analysis. (TIF) [file pone.0079804.s014.tif]

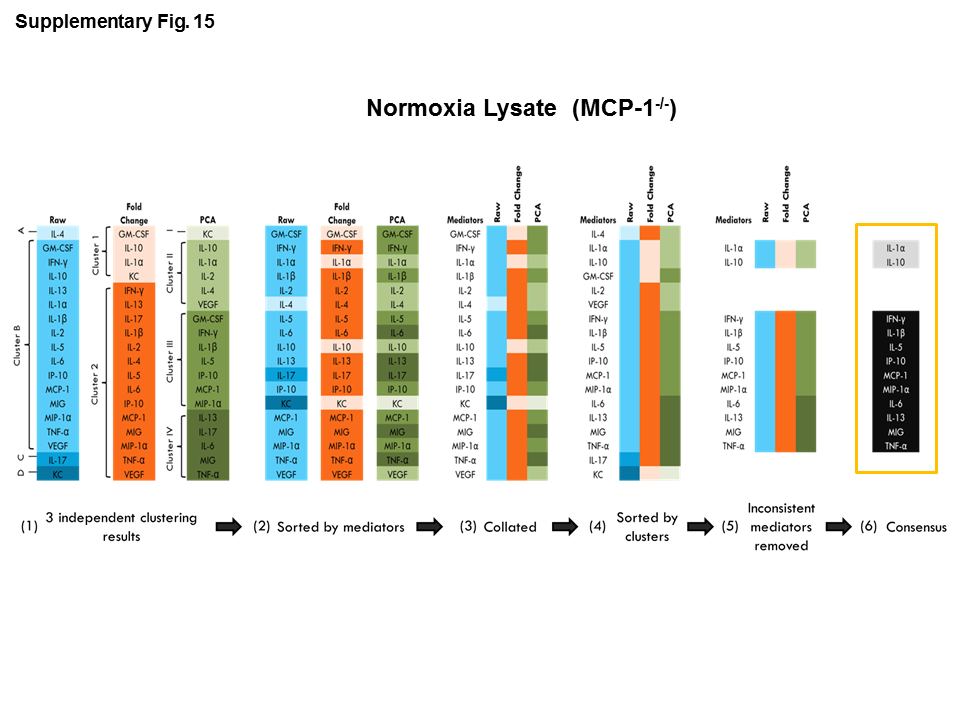

Supplement: Figure S15 — Meta-clustering analysis (see legend of Fig. S9) for normoxia lysates (MCP-1−/−): In Step 4 for this matrix, the rows were first sorted according to fold change cluster labels (orange), then by raw data cluster labels (blue), and finally by PCA clusters (green). In Step 5, all mediators from Cluster 1 (light orange) were also found in Cluster B (bright blue) and Cluster II (light green) and therefore, these three clusters were considered to be the same. Any mediators associated with Cluster 1, but not with both Cluster B and Cluster II were marked as inconsistent and discarded from the analysis. Clusters III (medium green) and IV (dark green) were both associated with Cluster B (bright blue) and Cluster 2 (bright orange). (TIF) [file pone.0079804.s015.tif]

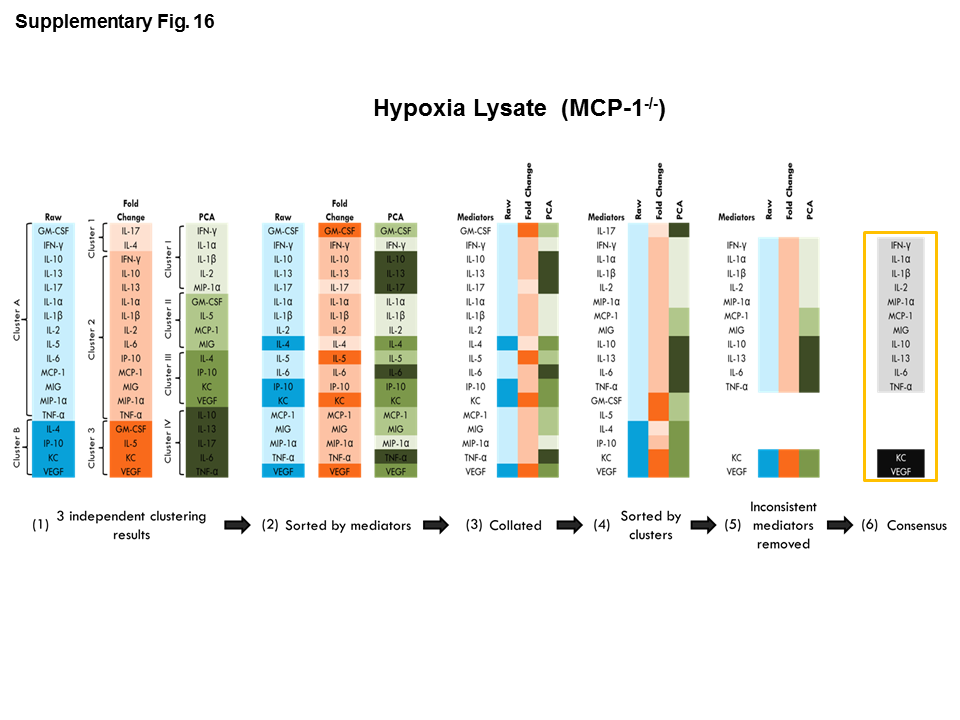

Supplement: Figure S16 — Meta-clustering analysis (see legend of Fig. S9) for hypoxia lysates (MCP-1−/−): In Step 4 for this matrix, the rows were first sorted according to raw data cluster labels (blue), then by fold change cluster labels (orange), and finally by PCA clusters (green). In Step 5, all mediators from Cluster B (medium blue) were also found in Cluster 3 (bright orange) and Cluster III (medium green) and therefore, these three clusters were considered to be the same. Any mediators associated with Cluster B, but not with both Cluster 3 and Cluster III were marked as inconsistent and discarded from the analysis. Clusters I (grey), II (light green), and IV (dark green) were all associated with Cluster A (light blue) and Cluster 1 (light orange). (TIF) [file pone.0079804.s016.tif]

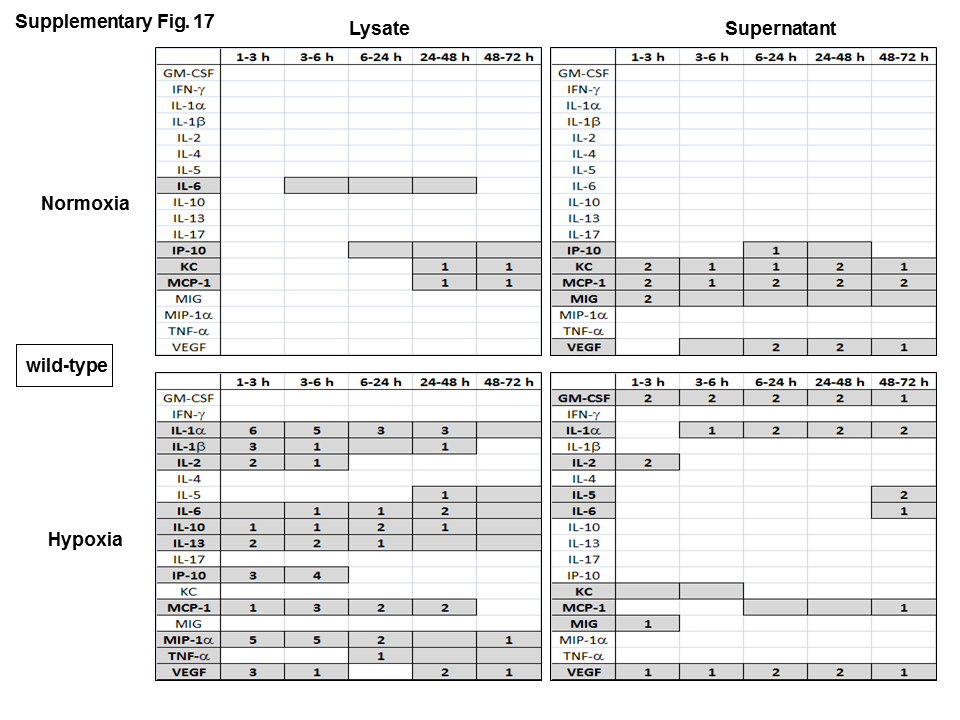

Supplement: Figure S17 — Dynamic Network Analysis (DyNA) of inflammatory mediators produced by normoxic and hypoxic mouse hepatocytes. Primary hepatocytes from wild-type mice were cultured under normoxic or hypoxic conditions (1–72 h) followed by measurement of cytokines/chemokines in both lysates and supernatants and lysates as described in the Materials and Methods . Gray boxes indicate that the mediator is statistically significantly different from its baseline value (no treatment [time = 1 h]; P<0.05) and the digits represent the number of connections resulting from the DyNAs during each of the following five time frames: 1–3 h, 3–6 h, 6–24 h, 24–48 h, and 48–72 h for both lysates and supernatants. (TIF) [file pone.0079804.s017.tif]

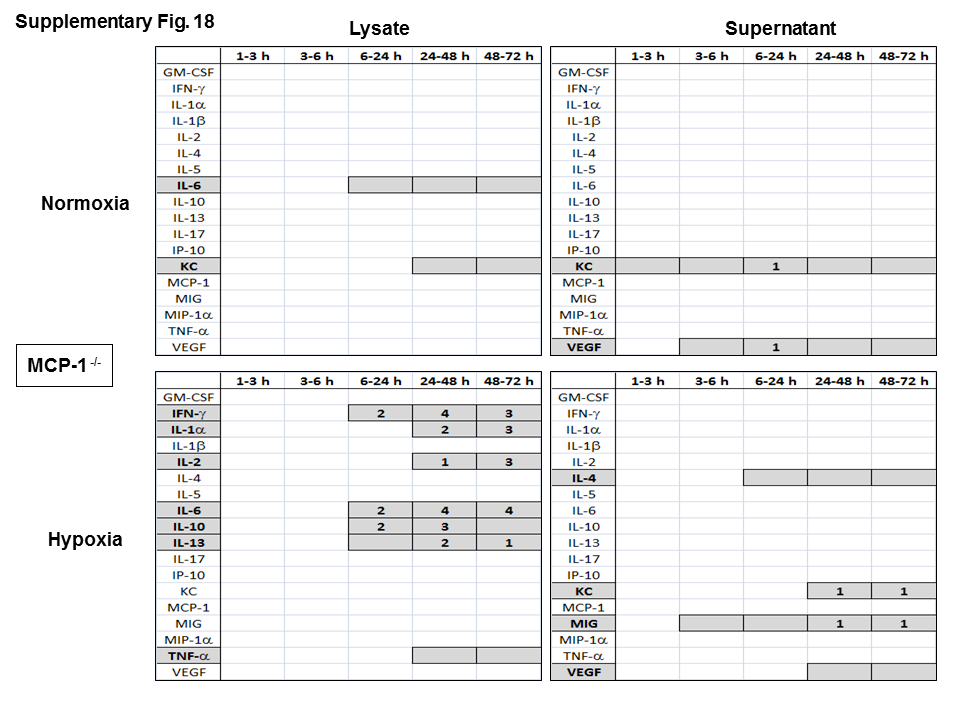

Supplement: Figure S18 — Dynamic Network Analysis (DyNA) of inflammatory mediators produced by normoxic and hypoxic mouse hepatocytes from MCP-1−/− mice. Primary hepatocytes from MCP-1−/− mice were cultured under normoxic or hypoxic conditions (1–72 h) followed by measurement of cytokines/chemokines in both lysates and supernatants and lysates as described in the Materials and Methods . Gray boxes indicate that the mediator is statistically significantly different from its baseline value (no treatment [time = 1 h]; P<0.05) and the digits represent the number of connections resulting from the DyNAs during each of the following five time frames: 1–3 h, 3–6 h, 6–24 h, 24–48 h, and 48–72 h for both lysates and supernatants. (TIF) [file pone.0079804.s018.tif]
